# Mapping the scarcity of data on antibiotics in natural and engineered water environments across India

**DOI:** 10.3389/frabi.2024.1337261

**Published:** 2024-02-12

**Authors:** Sasikaladevi Rathinavelu, Cansu Uluseker, Vikas Sonkar, Shashidhar Thatikonda, Indumathi M. Nambi, Jan-Ulrich Kreft

**Affiliations:** ^1^ Environmental Engineering Division, Department of Civil Engineering, Indian Institute of Technology Madras, Chennai, India; ^2^ School of Biosciences & Institute of Microbiology and Infection, University of Birmingham, Birmingham, United Kingdom; ^3^ Applied and Industrial Microbiology Lab, Department of Biotechnology, Indian Institute of Technology Madras, Chennai, India; ^4^ Department of Civil Engineering, Indian Institute of Technology Hyderabad, Sangareddy, Telangana, India

**Keywords:** antimicrobial resistance, antibiotic pollution, sewage, wastewater, environment, health risk, mitigation

## Abstract

Antimicrobial resistance is a growing public health concern, increasingly recognized as a silent pandemic across the globe. Therefore, it is important to monitor all factors that could contribute to the emergence, maintenance and spread of antimicrobial resistance. Environmental antibiotic pollution is thought to be one of the contributing factors. India is one of the world’s largest consumers and producers of antibiotics. Hence, antibiotics have been detected in different environments across India, sometimes at very high concentrations due to their extensive use in humans and agriculture or due to manufacturing. We summarize the current state of knowledge on the occurrence and transport pathways of antibiotics in Indian water environments, including sewage or wastewater and treatment plants, surface waters such as rivers, lakes, and reservoirs as well as groundwater and drinking water. The factors influencing the distribution of antibiotics in the water environment, such as rainfall, population density and variations in sewage treatment are discussed, followed by existing regulations and policies aimed at the mitigation of environmental antimicrobial resistance in India, which will have global benefits. Then, we recommend directions for future research, development of standardized methods for monitoring antibiotics in water, ecological risk assessment, and exploration of strategies to prevent antibiotics from entering the environment. Finally, we provide an evaluation of how scarce the data is, and how a systematic understanding of the occurrence and concentrations of antibiotics in the water environment in India could be achieved. Overall, we highlight the urgent need for sustainable solutions to monitor and mitigate the impact of antibiotics on environmental, animal, and public health.

## 1 Introduction

Antimicrobial resistance (AMR) poses a significant risk to global public health by complicating the treatment of various infections, raising mortality rates and inflating treatment expenses (World Health Organization, 2014). AMR has been described as a silent pandemic because it is not as noticeable and abrupt as natural disasters or outbreaks of disease yet it has spread and grown globally and caused an estimated 1.27 million (95% UI 0.911–1.71) deaths in 2019 (Antimicrobial Resistance Collaborators et al., 2022). The contributing factors to the rise of AMR include (but are not limited to) the use of antibiotics in human and veterinary medicine (whether appropriate or not), as well as their release into environmental compartments such as water and soil (Baquero et al., 2008; Finley et al., 2013; Serwecińska, 2020). Consequently, antibiotics present in the environment may under some conditions impart selective pressure on microorganisms, fostering the development and propagation of antibiotic-resistant bacteria (ARB) and antibiotic-resistance genes (ARGs) (Andersson and Hughes, 2011; Berendonk et al., 2015). At which concentrations antibiotics select for resistance in the environment is quite uncertain. Results from the growth of pure cultures in rich media have led to the concept of the minimal selective concentration (MSC) above which the resistant strain grows better than the otherwise identical sensitive strain and below which the resistant strain grows worse due to the fitness cost of resistance (Gullberg et al., 2011). However, fitness costs range from high to negligible or even negative and lower costs are selected for while trade-offs limit this reduction (Andersson and Levin, 1999; Harrison et al., 2015; Vogwill and Maclean, 2015; Loftie-Eaton et al., 2017; Cisneros-Mayoral et al., 2022; Delafuente et al., 2022). Slower growth, typical for most environments, can increase or decrease the efficacy of antibiotics (Greulich et al., 2015). Competition with other bacteria, almost universal in the environment, can reduce selection for resistance (Fang et al., 2023). Studies where an environmental sample with a diverse microbial community is added to a rich medium at various concentrations of an antibiotic (Stanton et al., 2020), are difficult to interpret as changes in resistance can be confounded by shifts in the community composition due to the shift from oligotrophic to copiotrophic conditions. Environmental observations of increases in resistance (Kurasam et al., 2022) can likewise be confounded. Nevertheless, it is fairly certain that the higher concentrations of (mixtures of) antibiotics found in hospital sewage select for resistance (Kraupner et al., 2021) while community wastewater may not be selective (Quintela-Baluja et al., 2021).

India holds a key position as one of the largest global producers and consumers of antibiotics, making it particularly susceptible to the detrimental effects of antibiotic pollution on environmental and public health (Chandy et al., 2013; Van Boeckel et al., 2014; Taneja and Sharma, 2019). The country has experienced a considerable rise in AMR cases, with numerous bacterial pathogens displaying resistance to multiple antibiotic classes (Laxminarayan and Chaudhury, 2016; Gandra et al., 2017). Environmental contamination by antibiotics in India can be traced back to their widespread use in human medicine, animal husbandry, and agriculture, as well as the release of effluents from pharmaceutical manufacturing facilities and sewage treatment plants (Larsson et al., 2007; Fick et al., 2009; Gothwal and Shashidhar, 2017a; Gothwal and Shashidhar, 2017b; Lübbert et al., 2017; Pérez-Rodríguez and Mercanoglu Taban, 2019).

Water environments are critical in the context of AMR, as they serve as reservoirs and transmission pathways for antibiotics, ARBs and ARGs. Antimicrobials, metals, and other biocides can cause stress that elicits stress responses, resulting in alterations in microbial communities, disrupting ecosystems and increasing rates of mutation or horizontal gene transfer. ARBs potentially expose both animals and humans to antibiotic-resistant infections, thus subsequently impacting ecosystems, animal populations, and human health (Rodriguez-Mozaz et al., 2015; Manaia et al., 2018). Various studies have reported the presence of antibiotics in several types of water bodies across India, including sewage systems, sewage treatment plants (STPs), surface waters, groundwater, and drinking water sources (Subedi et al., 2017; Taneja and Sharma, 2019). To assess the potential risks and develop mitigation strategies, it is crucial to understand the distribution and occurrence of antibiotics in these water bodies.

This review systematically analyses the existing knowledge on the presence, concentrations, and pathways of antibiotics in diverse water environments throughout India and an estimate of how scarce the data available to date are. It will explore factors that influence the distribution of antibiotics, such as population density, sewage or other point sources and variations in sewage treatment practices as well as precipitation and climate. An examination of the current regulations and policies aimed at mitigating environmental AMR in India will be provided, highlighting the challenges and gaps in their implementation. The review will also suggest directions for future research, including ways in which monitoring antibiotics in water can be scaled up, ecological risk assessment, and investigating strategies to prevent antibiotics from entering the environment. By synthesizing the current understanding of the occurrence and concentrations of antibiotics in India’s water environments, this review underscores the immediate need for sustainable solutions to tackle the environmental and public health challenges associated with antibiotics and AMR.

## 2 Methodology

Research papers were reviewed to gain an understanding of the levels and distribution of antibiotics in various water sources across India, including sewage systems, treatment plants, surface, and groundwater, as well as drinking water. Our research methodology was guided by the PRISMA (Preferred Reporting Items for Systematic Reviews and Meta-Analyses) guidelines to ensure a thorough and transparent approach (Page et al., 2021). This adherence involved following the prescribed steps and criteria set forth by PRISMA for systematic reviews and meta-analyses, ensuring consistency and rigour in our research process. We conducted our search using PubMed, Scopus, and Web of Science. The search string comprised of terms related to antibiotics, water environments and India: (“antibiotic*” OR “antimicrobial resistance”) AND (“water” OR “wastewater” OR “sewage” OR “sewage water” OR “surface water” OR “groundwater” OR “drinking water”) AND “India”.

The initial search yielded approximately 1600 articles in total. These articles were initially screened based on their titles and abstracts by two independent reviewers, in line with PRISMA’s selection process guidelines. The reviewers evaluated each article for its relevance to the current study’s objectives, as defined in the PRISMA guidelines. Domain knowledge was evaluated by considering the depth and breadth of information presented in each article, ensuring it aligns with the current study’s focus. This process ensured that the studies selected were relevant to our study’s objectives, as outlined in the PRISMA guidelines. After this preliminary screening, the full texts of potentially relevant articles were closely examined to establish their suitability for inclusion in our review. The search was confined to articles published in English between January 2000 and October 2023 to ensure recent and relevant data. Moreover, non-peer-reviewed reports or studies that did not pertain specifically to India were excluded.

**Figure 1:**
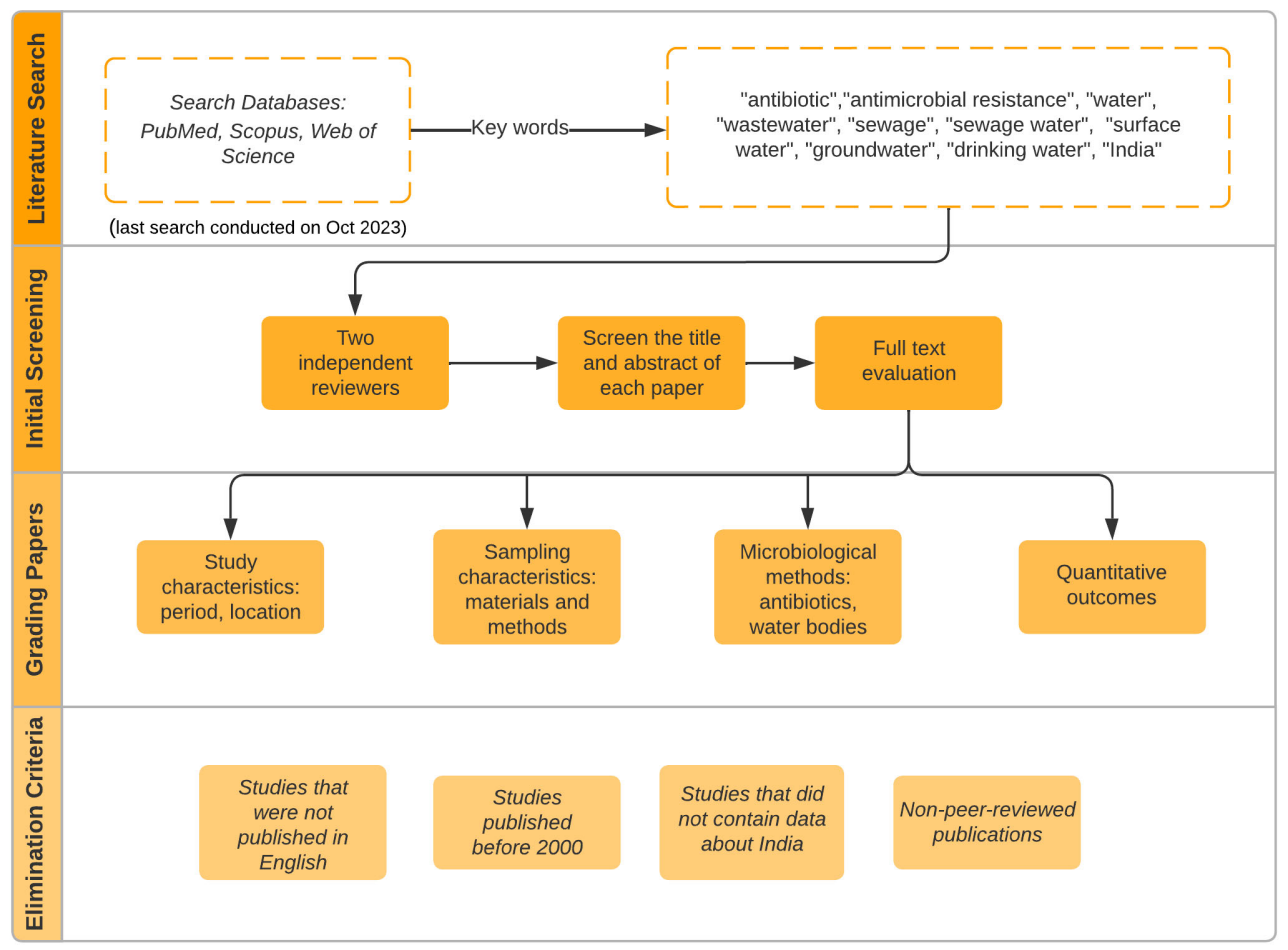
Schematic of the review methodology detailing the step-by-step process from database search to quality assessment.

The review methodology followed in this study is presented in **Figure 1**. The extracted data from the included studies were analysed according to the PRISMA data collection process and synthesis methods, which included study location and year, types of water matrices examined, antibiotics investigated, analytical methods used to quantify antibiotics, occurrence and concentration of antibiotics, and environmental factors (e.g., temperature, rainfall, population density) that could affect the distribution of antibiotics. Concentrations of antibiotics in water samples are given in µg L^-1^ and for sludge in µg g^-1^. For mapping the distribution and occurrence of antibiotics across different water environments in India, ArcMap software was employed. The quality of the included studies was assessed by evaluating the methodology. This assessment evaluated factors such as the sample collection procedure, analytical methods employed, statistical analyses performed, and whether potential confounding factors were considered.

## 3 Antibiotic classes and their properties

To effectively analyse the distribution and impact of antibiotics in water environments across India, it is useful to understand the specific characteristics of the antibiotics frequently detected. These characteristics include the class to which an antibiotic belongs, its molecular weight and chemical structure, here only given by its formula (**Table 1**). These properties play a role in determining how an antibiotic interacts with the environment. They can thus contribute to the promotion and maintenance of antibiotic resistance. Other factors, such as polarity, octanol-water partition coefficient, sorption, and degradation rates under prevailing environmental conditions, are also important in understanding the behaviour and fate of antibiotics in aquatic ecosystems (Harrower et al., 2021).

**Table 1:** Some of the antibiotics most commonly detected in India’s water bodies, including the class of each antibiotic, its molecular mass and chemical formula (PubChem Compound Summary, 2023).

Antibiotic	Class	Molecular Mass (g/mol)	Chemical Formula	Reference
Amoxicillin	Penicillin	365.41	C_16_H_19_N_3_O_5_S	(PubChem Amoxicillin, 2023)
Ciprofloxacin	Quinolone	331.34	C_17_H_18_FN_3_O_3_	(PubChem Ciprofloxacin, 2023)
Azithromycin	Macrolide	749	C_38_H_72_N_2_O_12_	(PubChem Azithromycin, 2023)
Doxycycline	Tetracycline	444.43	C_22_H_24_N_2_O_8_	(PubChem Doxycycline, 2023)
Erythromycin	Macrolide	733.94	C_37_H_67_NO_13_	(PubChem Erythromycin, 2023)
Metronidazole	Nitroimidazole	171.15	C_6_H_9_N_3_O_3_	(PubChem Metronidazole, 2023)
Vancomycin	Glycopeptide	1449.22	C_66_H_75_C_l2_N_9_O_24_	(PubChem Vancomycin, 2023)
Gentamicin	Aminoglycoside	477.58	C_21_H_43_N_5_O_7_	(PubChem Gentamicin, 2023)
Tetracycline	Tetracycline	444.43	C_22_H_24_N_2_O_8_	(PubChem Tetracycline, 2023)
Trimethoprim	Dihydrofolate reductase inhibitor	218.24	C_14_H_18_N_4_O_3_	(PubChem Trimethoprim, 2023)
Sulfamethoxazole	Sulfonamide	253.28	C_10_H_11_N_3_O_3_S	(PubChem Sulfamethoxazole, 2023)

These antibiotics were selected based on their frequent usage in human medicine and animal husbandry in India, as well as their documented occurrence in various types of water bodies. For this reason, this list may be biased towards compounds that are often analysed.

The class of an antibiotic can affect the spectrum of bacteria it targets, its mode of action and its mode of resistance. For instance, antibiotics belonging to the penicillin class are more effective against Gram-positive than Gram-negative bacteria and work by inhibiting bacterial cell wall synthesis (Calderón and Sabundayo, 2007; Kapoor et al., 2017). In contrast, aminoglycosides like gentamicin target Gram-negative bacteria and inhibit protein synthesis. A lower molecular mass and thus smaller size increases the diffusivity of an antibiotic, more rapidly dispersing it over the short range of less than a millimetre at which diffusion is effective (Jackson et al., 2018).

Beyond the chemical formula, which provides insights into the elemental composition of an antibiotic, its specific chemical structure plays a vital role. Different compounds with the same elemental composition can possess unique structures, leading to diverse behaviours in the environment. These structures offer clues into the antibiotic’s stability, reactivity, and potential transformation pathways in various environmental settings (Kümmerer, 2009). Degradation and sorption rates of antibiotics are critical in understanding the persistence and mobility of these compounds in the environment. A higher degradation rate implies quicker breakdown, while the sorption rate indicates the tendency of the antibiotic to bind to environmental matrices such as sediments or organic matter (Kümmerer, 2009). Knowing these rates aids in predicting the potential accumulation and distribution of antibiotics in water systems.

The commonly detected antibiotics in **Table 1** represent a wide range of classes and have different characteristics that influence their persistence, distribution, and effects in the environment. For example, tetracyclines and sulfonamides are known for their persistence in the environment due to their chemical stability, while penicillins and other β-lactam antibiotics are relatively unstable as they can be hydrolysed and thus degrade faster. On the other hand, the quinolones and macrolides show intermediate stability (Kümmerer, 2009; Carvalho and Santos, 2016). Further sections of this review will delve into the occurrence of these antibiotics in various water environments across India, the analytical methods used for their detection, and the implications of their presence for antibiotic resistance development and public health.

## 4 Sources and factors contributing to the distribution of antibiotics in the environment

**Figure 2:**
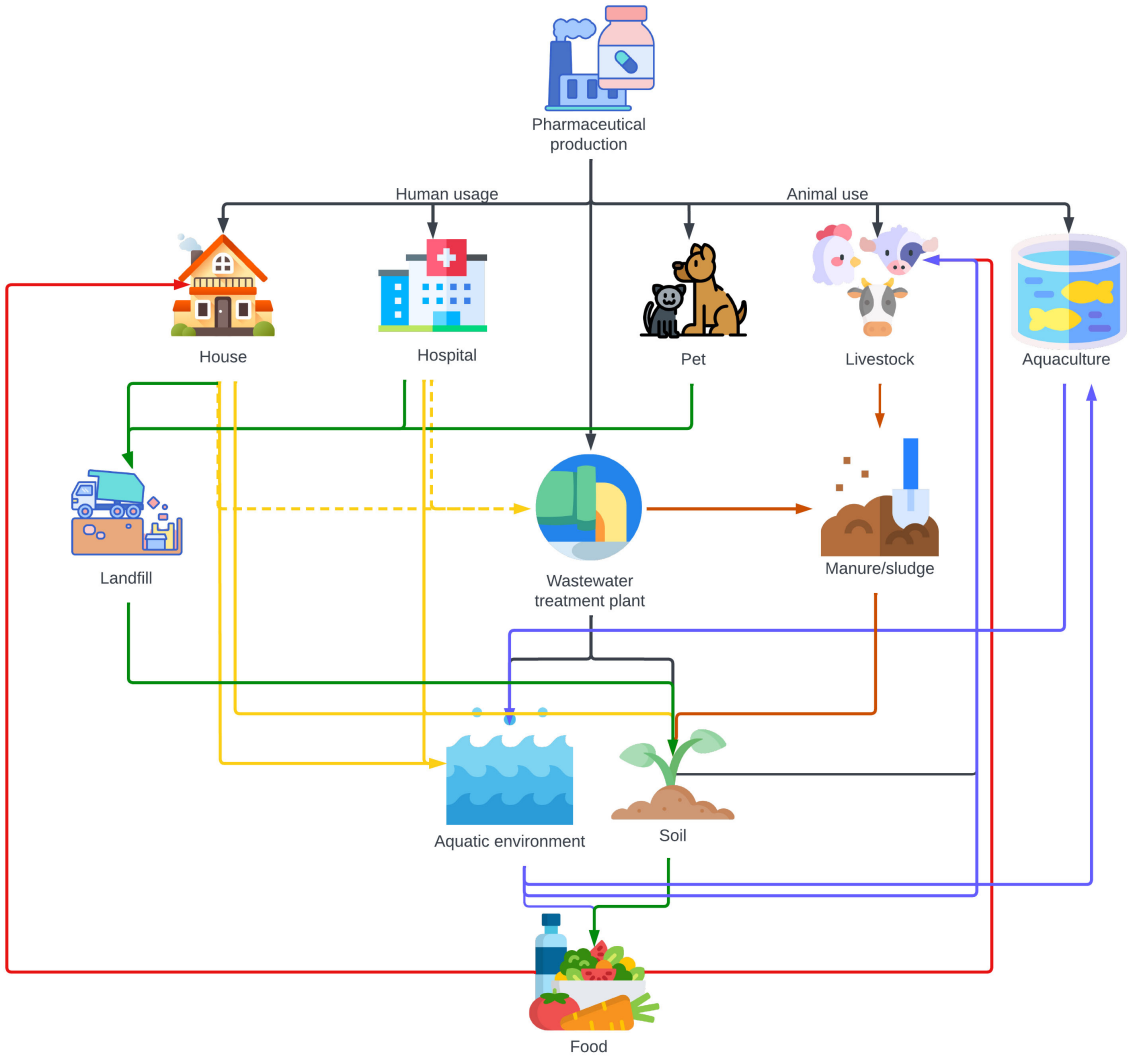
Illustration of the environmental pathways through which antibiotics are transported, directly from pharmaceutical production or indirectly via consumption by humans and veterinary applications. Waste from pharmaceutical factories manufacturing antibiotics is typically discharged into wastewater treatment plants. Produced antibiotics are then consumed by humans in households and hospitals and by animals, including pets, livestock, and aquaculture settings. Interestingly, in areas like India with limited sewage infrastructure, antibiotics from households and hospitals predominantly bypass STPs and directly enter the aquatic ecosystem and infiltrate soil. Only a minor portion (indicated by dotted lines) of human usage reaches STPs via sewage systems. The pathways are colour-coded: black signifies the direct discharge from production; green denotes the route leading to landfills; brown highlights the path via manure and sludge; blue represents aquatic dispersal; yellow captures both direct and indirect human-induced effects via STPs; and red pinpoints the direct incorporation into food.

The major sources of antibiotics in Indian environmental matrices are the waste generated from human and animal consumption and the pharmaceutical companies producing or formulating antibiotics. **Figure 2** represents the environmental compartments receiving antibiotics directly from production facilities or indirectly through veterinary use and human consumption. Arrows show the main pathways through which they are transported between these segments and the aquatic ecosystems. The consumption of antibiotics by humans and animals is the major contributor to antibiotic pollution in most locations, apart from sites of industrial production.

The environmental impact of waste dumps, landfills, manure, wastewater, and sewage systems, and rainfall is multifaceted and interconnected. Waste dumps, which are unregulated areas where waste is simply discarded, pose a significant environmental threat. They are different from official landfill sites, which are managed areas designated for waste disposal. Both can leach contaminants into the surrounding soil and groundwater, particularly during rainfall. Similarly, manure from livestock treated with antibiotics contributes considerably to soil and water contamination. When this manure is used as fertilizer, antibiotics can seep into the ground and nearby water bodies, a process further exacerbated by rainfall which facilitates the runoff of these contaminants. Wastewater and sewage systems refer to the infrastructure used for the collection, transport, treatment, and disposal of sewage and other wastewater. This includes both domestic and industrial wastewater, which often contains pharmaceuticals. These systems play a crucial role in the dissemination of antibiotics and other pollutants into aquatic environments. Inadequate treatment of these wastewater can result in the release of active pharmaceutical ingredients into rivers, lakes, and oceans. Rainfall again acts as a facilitator, increasing the flow of untreated or partially treated sewage into natural water bodies, thereby spreading these contaminants over a wider area and posing a substantial risk to environmental health and biodiversity, although the increased dilution through rainfall may lower the risk.

In India, antibiotics are listed under schedule H prescription drugs, which cannot be sold without a prescription from a qualified medical practitioner. Unrestricted over-the-counter sales in retail pharmacies combined with self-prescription for acute illness is a common practice (Blanchard et al., 2021; Kotwani et al., 2021). Self-prescription of antibiotics is mostly for viral-associated fever, cough, cold and acute diarrhoea, which are self-limiting. About 64% of patients in India self-prescribe antibiotics, which can be attributed to a lack of stringent rules and monitoring and a lack of awareness as well as commercial interests and economic growth enabling easy access to antibiotics (Subedi et al., 2017; Kotwani et al., 2021). A rise of 67% in antibiotic consumption in BRICS countries (Brazil, Russia, India, China, and South Africa) is expected by 2030. High population density and easy access to healthcare inadvertently increase the antibiotic consumption rate. A cross-sectional analysis based on the private sector drugs sales data set of PharmaTrac (Koya et al., 2022) reported a total defined daily dose of 5071 million in India of which 47.1% are unapproved formulations. Further, depending on the class of antibiotics consumed, 40-90% are excreted as active forms by humans, which end up in the sewage. **Table 2** presents the antibiotics with their concentration reported in sewage across India.

**Table 2:** Antibiotics of class sulfonamide and dihydrofolate reductase inhibitors reported in sewage across India.

Antibiotic	Concentration (µg L^-1^)	Region	Location	Reference
**Sulfamethoxazole**	1.19	Southern	Chennai	(Subedi et al., 2015)
	0.254			
	0.07		Coimbatore	
	0.298	Central	Patna	
	0.538	Eastern	Kolkata	(Shimizu et al., 2013)
**Trimethoprim**	0.0948	Southern	Chennai	
	0.0574			
	0.0143		Coimbatore	
	0.063	Central	Patna	
	51.82	Northern	Ghaziabad	(Singh et al., 2014)
	5.11		Lucknow	

### 4.1 Antibiotics in wastewaters and treatment plants

Urban wastewater is an important source of antibiotics in aquatic environments. Treating wastewater reduces antibiotic concentrations somewhat but does not eliminate them (see below). It is widely observed that hospital wastewaters carrying antibiotics drain into domestic sewer networks. Prior to discharge, hospital wastewater is rarely treated. Mixed wastewater (domestic mixed with hospital wastewater) is treated at STPs in metropolitan cities. However, the majority of small cities and towns lack a wastewater treatment facility resulting in direct discharge of the mixed sewage into environmental matrices. Diwan et al. (2010) studied the antibiotic concentrations in hospital wastewater at the point of discharge and downstream in a sewage network in Madhya Pradesh (Northern region of India) (**Table 3**). They found a positive correlation between antibiotic prescription and concentration in the wastewater. Surprisingly, the concentrations of ofloxacin, ciprofloxacin, norfloxacin and levofloxacin were higher downstream than in the hospital effluent (Diwan et al., 2010). This could be due to temporal fluctuations in concentrations or volume flows, also mixing in the sewer network may have been incomplete. Since upstream concentrations and flows were not measured, a mass balance cannot be calculated, which highlights the importance of effective study design and reporting (Hassoun-Kheir et al., 2020; Hassoun-Kheir et al., 2021).

**Table 3:** Fluoroquinolone antibiotics reported in Ujjain hospital wastewater and the sewer it drains into (Madhya Pradesh, Northern region of India) (Diwan et al., 2010).

Antibiotic (µg L^-1^)	Point of Discharge	100 m downstream from the discharge point
**Ofloxacin**	4.5	7.5
**Ciprofloxacin**	218.3	236.6
**Norfloxacin**	6.4	29.6
**Levofloxacin**	5	8.8

**Figure 3:**
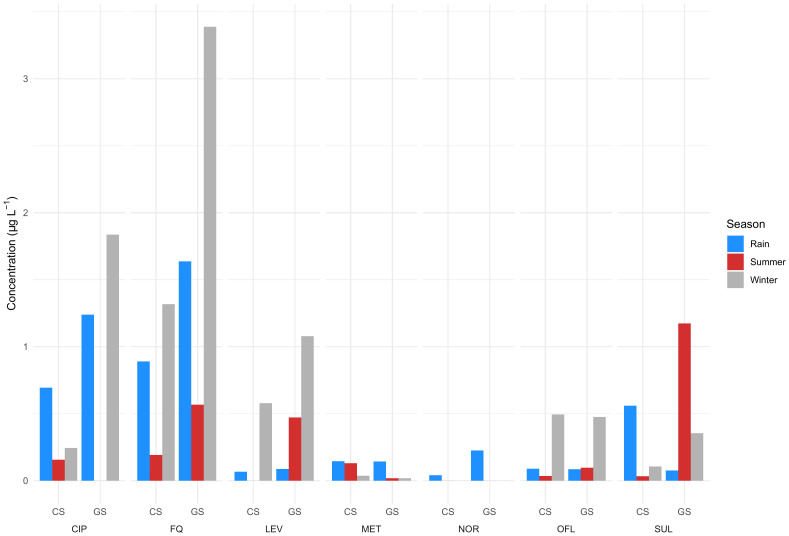
Antibiotics observed during winter, rainy, and summer seasons in two hospital effluents. CS, Composite sample; GS, Grab sample; CIP, Ciprofloxacin; LEV, Levofloxacin; OFL, Ofloxacin; NOR, Norfloxacin; FQ, Fluoroquinolones; MET, Metronidazole; SUL, Sulfamethoxazole (Diwan et al., 2013). Data can be found in **Supplementary Table S1**.

Seasons play an important role in determining the antibiotic consumption rate and therefore, the detection frequency and concentration in the wastewater, there may also be changes in the volume of wastewater produced per person. A study by Diwan et al. (2013) on temporal and seasonal variations of eight antibiotics of four different classes in two hospital effluents suggests that typically, antibiotic concentrations were highest in the winter followed by rainy and summer seasons, although this pattern is not followed by all antibiotics and sample types (**Figure 3**; **Supplementary Table S1**). This could be attributed to a high consumption rate due to the high number of infections in winter and rainy seasons and therefore high concentration of hospital effluents. Low concentration in summer was attributed to high microbial activity and environmental factors enabling photodegradation (due to intense sunlight) and degradation (due to high temperature). In addition, the concentrations can also be influenced by sewage flow rate. It was also estimated that 89 × 10^-3^, 1 × 10^-3^ and 25 × 10^-3^ µg L^-1^ day^-1^ of fluoroquinolones, metronidazole and sulfamethoxazole antibiotics are released by a 100-bed hospital into the sewer network (Diwan et al., 2013). Further, concentrations in grab samples were higher than in the composite samples (**Figure 3**; **Supplementary Table S1**). Corresponding samples from two hospital effluents were pooled so the reported values should be regarded as averages (**Supplementary Table S1**). The data confirms that the time and method of sampling influence the frequency and concentrations of antibiotics detected in the samples. In another study, Diwan et al. (2010) observed relatively high concentrations and a greater number of antibiotics in the afternoon compared to the forenoon, which could be attributed to consumption and excretion time patterns. While the study dates back more than a decade, hospital wastewater treatment has not improved despite a growing economy and infrastructure development and the situation is still similar across Indian cities.

Further, ineffective wastewater management and illegal discharge of treated and untreated wastewater into sewer networks by pharmaceutical industries remains a problem, despite the discharge rules and regulations in India limiting BOD, COD, TSS NH_3_-N, P, and others but not antibiotics (**Supplementary File S1**). Lübbert et al. (2017) reported the following concentrations of antibiotics in the sewer networks of Patancheru industrial area, a pharmaceutical hub in Hyderabad (Southern India) in μg L^-1^: moxifloxacin (7.1–694.1), levofloxacin (BDL–2.2), ciprofloxacin (BDL–9.4), ampicillin (BDL–29.1), linezolid (13.6–37), clarithromycin (BDL–13.5) and doxycycline (BDL–14.9) (Lübbert et al., 2017). Ultimately, sewage carrying antibiotics from different sources either ends up in an STP or is discharged into nearby surface waters from where it may enter other environmental matrices. In Chennai, South India, Arun et al. (2022) investigated the presence of 18 antibiotics in water sources and STPs. The detection frequencies of fluoroquinolones (up to 100%) were high in STPs. The Buckingham Canal showed higher antibiotic concentrations than the Adyar and Cooum rivers, likely due to direct sewer outfalls, solid waste dumping, low dilution, and low flow in the narrow and stagnant Buckingham Canal. The research highlights the potential risks due to antibiotic resistance in surface waters and suggests that direct sewage outlets are significant contributors to water contamination in Chennai.

**Figure 4:**
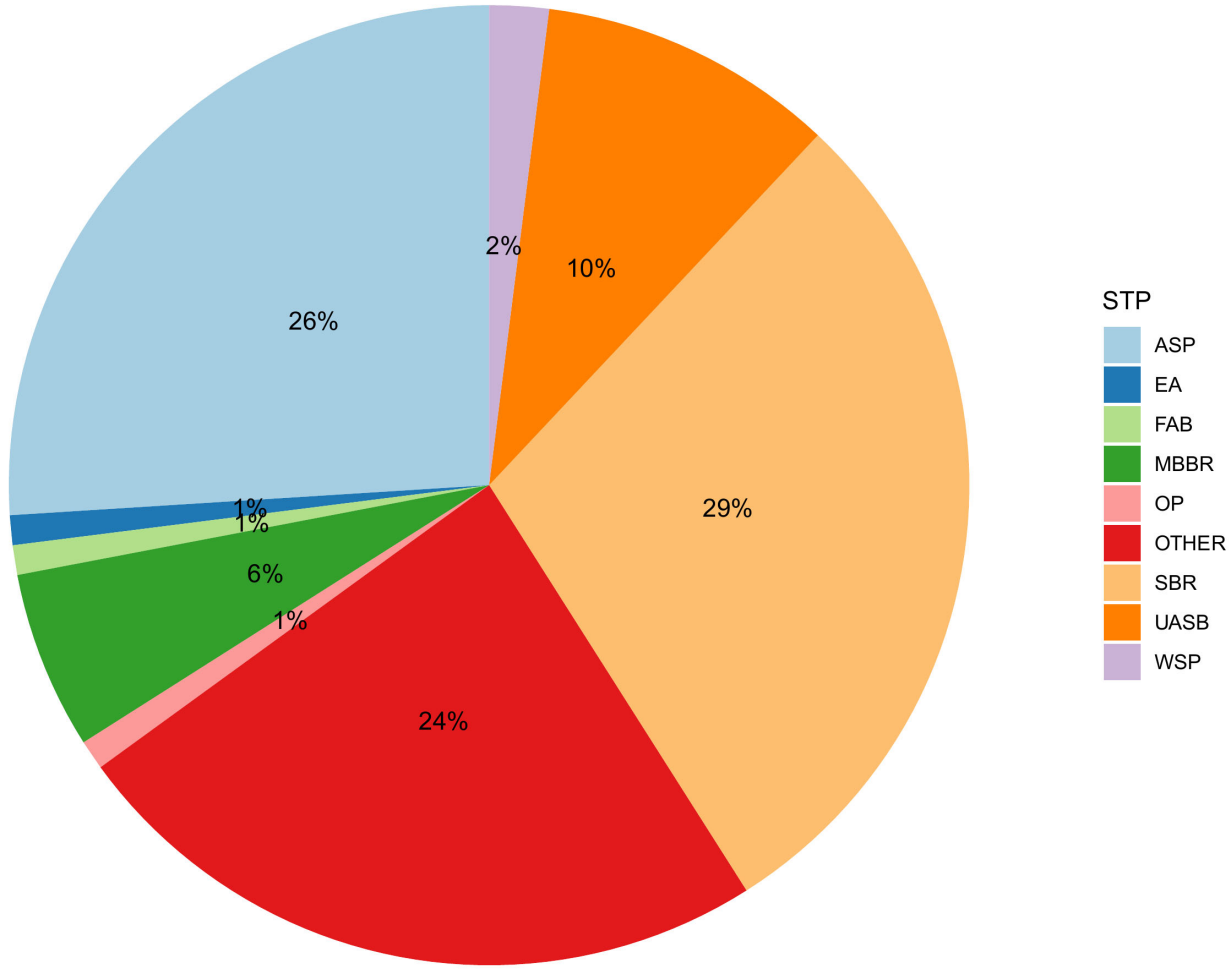
Distribution of different wastewater treatment processes across India (Reproduced from National Inventory of Sewage Treatment Plants) (Central Pollution Control Board, 2021). ASP, Activated sludge process; EA, Extended aeration; FAB, Fluidized aerobic bed reactor; MBBR, Moving bed biofilm reactor; OP, Oxidation Pond; SBR, Sequencing batch reactors; UASB, Upflow anaerobic sludge blanket; WSP, Waste stabilization pond. SBR is the most commonly employed (490 STPs) with a treatment capacity of 10,638 MLD followed by ASP (321 STPs, 9486 MLD), MBBR (201 STPs, 2032 MLD) and UASB (76 STPs, 3562 MLD).

The drainage and sewerage systems and STPs in India are not built as fast as the cities grow and are poorly regulated and monitored, as a result a large fraction of wastewater is not treated (Central Pollution Control Board, 2021). The sewage generated in urban centres alone stands at 72,368 million litres per day (MLD). This is much higher than the total wastewater treatment capacity of 36,668 MLD of which the operational capacity is 26,869 MLD and the utilization capacity is even lower at 20,235 MLD. Out of 1,631 STPs, 1,093 are functional and only 578 (12,200 MLD) comply with the discharge norms. It is estimated that 63 – 72% of the accounted sewage is discharged without treatment (Central Pollution Control Board, 2021). Clearly, the existing treatment capacity is not enough to cater for the 1.4 billion population with a wastewater generation capacity of 148 litres per capita per day (80% of 185 LPCD). The most employed wastewater treatment technologies include sequencing batch reactors (SBR), activated sludge process (ASP), upflow anaerobic sludge blanket (UASB) reactors and moving-bed biofilm reactors (MBBR) as well as a large variety of other technologies such as extended aeration (EA), fluidized aerobic bed (FAB) reactors, oxidation ponds (OP) and waste stabilization ponds (WSP). **Figure 4** presents the percentage distribution of different wastewater treatment processes across India (Central Pollution Control Board, 2021).

The influent, effluent, and sludge concentrations of antibiotics in STPs treating domestic sewage and employing ASP in Udupi, Manipal, Coimbatore and Chennai in the Southern region as well as Saidpur and Beur in the Northern region is presented in **Supplementary Table S1** (Subedi et al., 2015; Subedi et al., 2017; Arun et al., 2022). The influent and effluent of two STPs in Western India (locations not specified), employing a facultative aerated lagoon and cyclic activated sludge process followed by chlorination, carried antibiotics such as azithromycin, ciprofloxacin, levofloxacin, and sulfamethoxazole. The differences in inlet antibiotic concentration between the two STPs were speculated to arise from spatial variation in antibiotic usage, prescription patterns in the regions where the STPs were located and zonal planning. STP-1 was located in an area with relaxed regulations where illegal discharge of wastewater by industries and hospitals could occur. STP-2 was located in a better planned zone with strict regulations. The amoxicillin concentration was higher in the influent than the effluent of Vasantkunj STP (Delhi) in samples collected between October and March (years of study not reported) during 5 sampling campaigns, which again confirm the seasonal influence on antibiotic consumption and detection in sewage (Mutiyar and Mittal, 2013) (**Table 4**).

**Table 4:** Amoxicillin reported in the influent and effluent of Vasankunj STP (Delhi) during 5 sampling campaigns between October and March (Mutiyar and Mittal, 2013).

Sampling Campaign	Influent (µg L^-1^)	Effluent (µg L^-1^)
1	0.0614– 0.065	0.0531 – 0.058
2	0.0882 – 0.1408	BDL* – 0.0625
3	0.0539 – 0.0981	0.0229 – 0.0233
4	BDL* – 0.0367	BDL*
5	0.0644 – 0.1726	0.023 – 0.0521

*BDL, Below Detection Limit.

**Figure 5:**
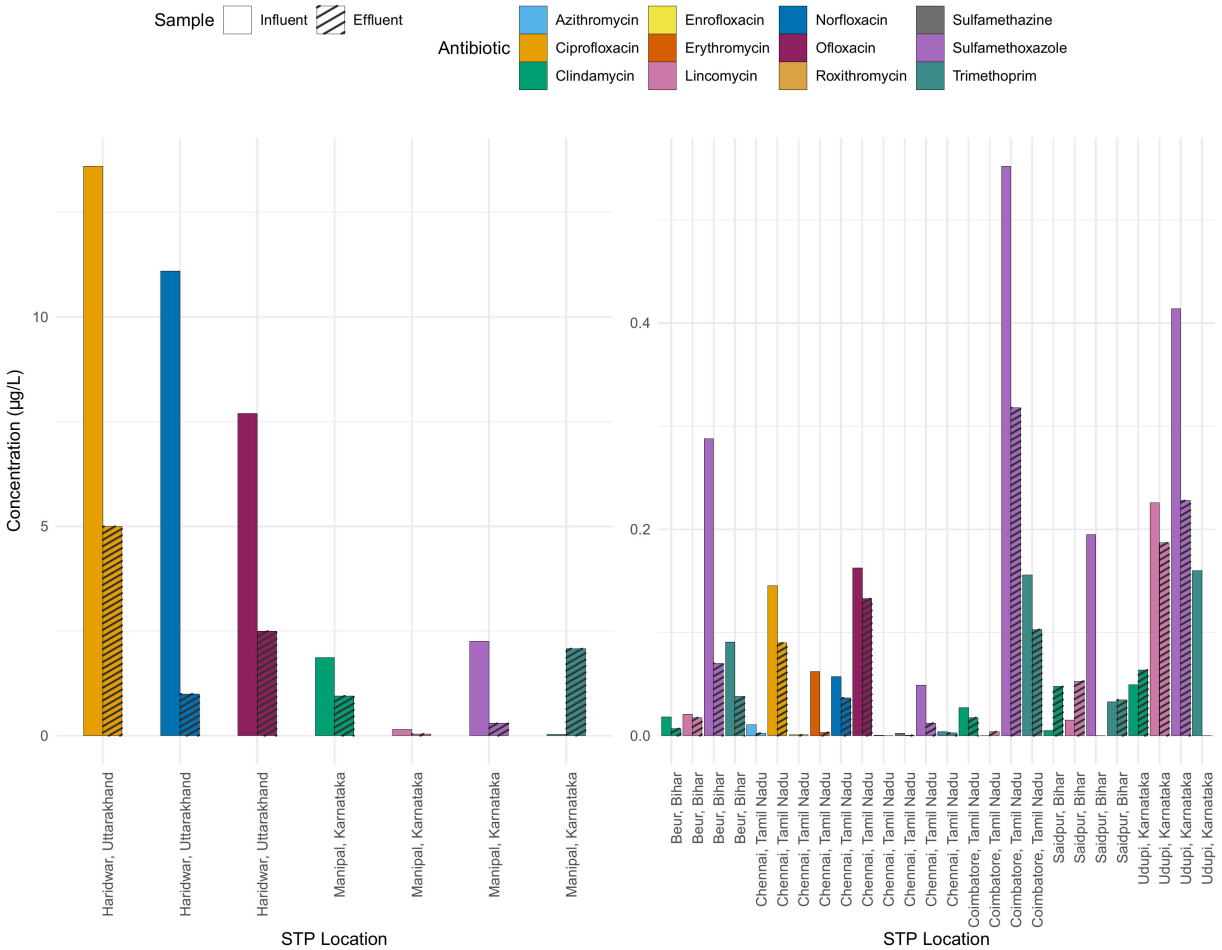
Comparison of antibiotic concentrations in influent and effluent across various STPs. Each antibiotic is represented by a distinct colour, with the plain bars denoting influent concentration and hatched bars indicating effluent concentration. Note the two different scales for the y-axis representing the concentration in µg L^-1^, while the x-axis lists the STP locations.

Seasonal changes also influence the influent antibiotic concentrations with the highest concentrations in winter followed by summer and monsoon, largely owing to seasonal infections in winter and dilution during monsoon (Mohapatra et al., 2016). Some examples: The amoxicillin concentration in the effluent of Vasantkunj STP (Delhi, North India) employing extended aeration was up to 0.0625 µg L^-1^ (Mutiyar and Mittal, 2013). Ciprofloxacin in the effluent of an STP in Nagpur (West India) employing MBBR was 438 µg L^-1^ (Saxena et al., 2021). An STP in Mangalore (Karnataka, Southern India) employing UASB discharges up to 0.029 µg L^-1^ of trimethoprim, sulfamethoxazole, clindamycin and 0.31 µg L^-1^ of lincomycin (Subedi et al., 2017). Clearly, the antibiotic concentrations reported in the effluents and sludge across India further confirm the inefficiency of existing STPs to remove the antibiotics (**Figure 5**). The sludge and treated effluent from STPs are also used for fertilization or irrigation of agricultural fields. For instance, effluent from three STPs in Karnataka (South India) treating mixed wastewater (hospital and domestic) by the extended aeration activated sludge process is reused for agriculture post disinfection. However, the effluents during pre-monsoon, monsoon and post monsoon months carried sulfamethoxazole, trimethoprim, chloramphenicol, erythromycin, and ampicillin at varying concentrations. The effluent from another STP, draining into underground drainage post treatment, also carried these antibiotics (Prabhasankar et al., 2016). Similarly, another STP that employs ASP in Delhi (North India) also discharges secondary effluent with sulfamethoxazole (200 µg L^-1^) into an irrigation canal. Moreover, during rainfall events, STPs may receive diluted wastewater above the holding and treatment capacity, resulting in discharge without treatment or partial treatment that introduces antibiotics into the receiving waters.

**Figure 5** shows the concentrations of various antibiotics in different STPs in influent (input) and effluent (output). The difference between the influent and effluent concentrations indicates the effectiveness of the STP in reducing the antibiotic concentration. In some STPs, specific antibiotics show significant reductions from influent to effluent, suggesting effective treatment processes, for example ciprofloxacin. In the STP in Manipal, the concentration of trimethoprim in the effluent is higher than in the influent, which is difficult to understand.

### 4.2 Antibiotics in surface water bodies and groundwater

**Figure 6:**
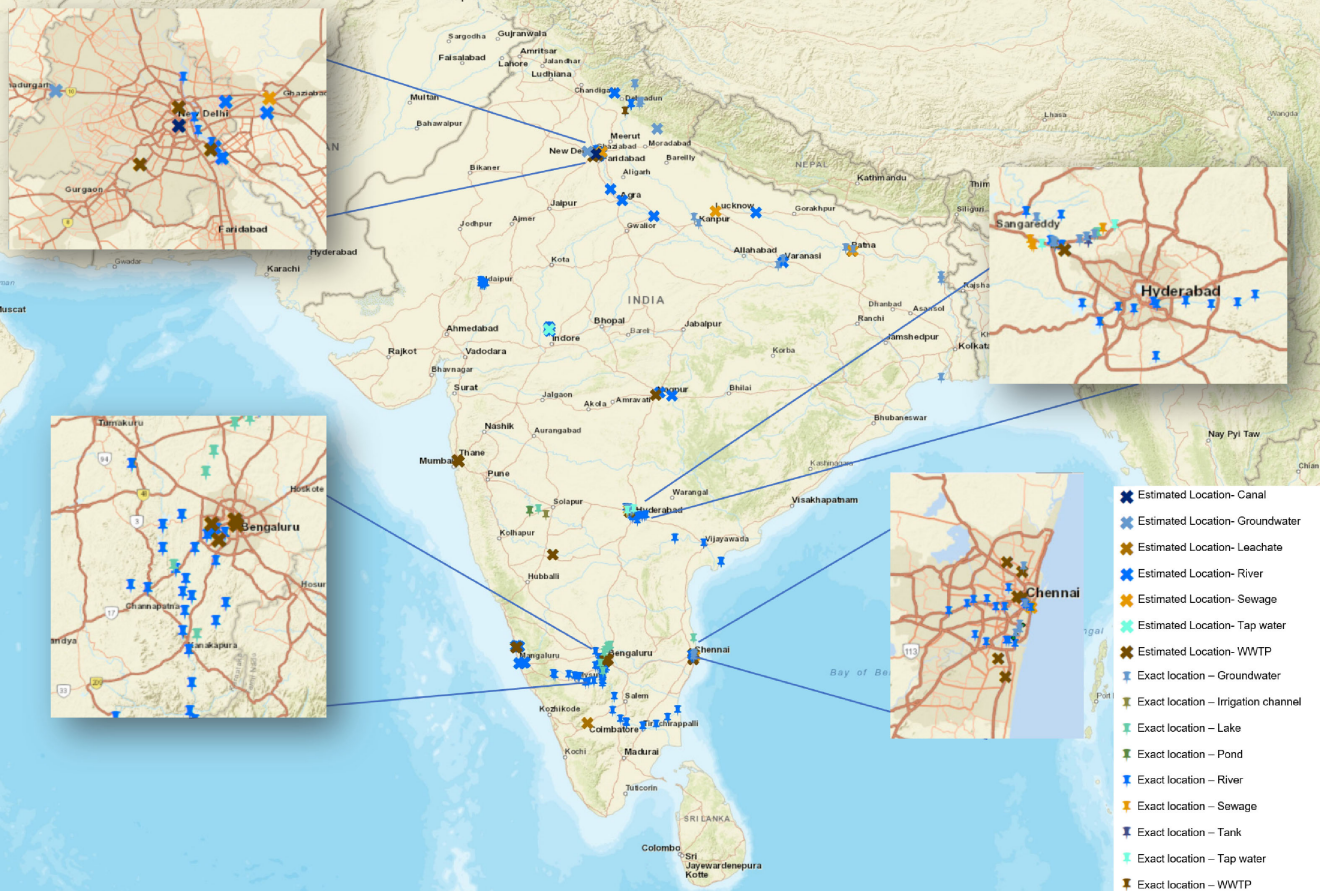
Geographic distribution of antibiotic monitoring studies in Indian water environments. Each mark on the map represents a location that has been studied. The pushpin indicates studies where exact geographical coordinates were provided, while the cross represents studies where the location had to be estimated based on available information in the text. Different water sources are represented with specific colour codes: blue for surface waters (rivers, lakes, reservoirs), groundwater and tap water, and brown and orange for sewage and STPs. The density and spread of the dots underscore the limited extent and coverage of antibiotic monitoring across the country. While some regions exhibit a relatively higher density of studies, others are hardly or not at all explored, indicating areas for future investigations.

Direct discharge of untreated or poorly treated sewage into nearby water matrices due to poor wastewater management is common both in urban and rural regions across India. The majority of STPs are constructed along riverbanks for easy discharge of wastewater after treatment. Biological treatment technologies are only partially removing antibiotics (**Figure 5**); thus, effluents ultimately pollute the receiving water bodies. **Figure 6** presents the location of water bodies such as wastewaters, STPs, canals, rivers, irrigation channels, lakes, ponds, tanks, and ground water contaminated with antibiotics across India. Anthropogenic activities such as the discharge of untreated domestic and industrial waste and sewage into river basins is also recorded. In North India, four out of eight studies are on the Yamuna River (tributary of River Ganga) while the other four are on the Ganga and its other tributaries. Various concentration of metronidazole, sulfamethoxazole, sulfanilamides, trimethoprim, chloramphenicol, cephalexin, cefixime, cefuroxime, ampicillin, amoxicillin, penicillin, kanamycin, tobramycin, tetramycin, streptomycin, amikacin, netlimycin, neomycin, gentamycin, lincomycin, erythromycin, azithromycin, ofloxacin, doxycycline, ciprofloxacin, levofloxacin, moxifloxacin, getifloxacin, sparfloxacin, norfloxacin, and enrofloxacin have been detected across different seasons (Mutiyar and Mittal, 2014; Jha et al., 2017; Lapworth et al., 2018; Velpandian et al., 2018; Sharma et al., 2019; Biswas and Vellanki, 2021; Singh and Suthar, 2021; Wilkinson et al., 2022). Antibiotics such as getifloxacin (0.48 µg L^-1^), cefuroxime (1.7 µg L^-1^) and sparfloxacin (2.09 µg L^-1^) were detected in the Yamuna only in winter, which again confirms the seasonal influence on antibiotic use and detection in environmental matrices (Mutiyar and Mittal, 2014). In South India, the Musi River is the most studied, which was declared to be one of the most polluted rivers by the CPCB of India (Central Pollution Control Board, 2017). Hyderabad, the Musi receives effluents from four STPs and untreated wastewater from ten canals, run-off from agricultural areas and waste is dumped along the riverbank. Several fluoroquinolones (ciprofloxacin, enrofloxacin, norfloxacin, pefloxacin, difloxacin, lomefloxacin, ofloxacin, levofloxacin, moxifloxacin) with ciprofloxacin as high as 5,528 µg L^-1^, macrolides (clarithromycin), penicillins (ampicillin), tetracyclines (doxycycline), sulfonamides (sulfamethoxazole), and others (linezolid, trimethoprim) have been detected throughout the stretch (Fick et al., 2009; Gothwal and Shashidhar, 2017a; Lübbert et al., 2017). Several antibiotics at various concentrations have been reported in rivers Krishna, Cauvery, Arkavathi, Swarna and Nethravathi (South India), Kshipra, Nag, Pili (Central India) and Ahar (West India) (Archana et al., 2016; Diwan et al., 2018; Williams et al., 2019; Gopal et al., 2020; Hanna et al., 2020; Joshua et al., 2020; Gopal et al., 2021; Renganathan et al., 2021). Water from rivers across India is widely used for both drinking and irrigation, vital for producing food.

Other water matrices such as lakes, ponds, tanks, canals, and ground water are also contaminated with antibiotics (**Figure 6**) as a result of direct discharge of effluents from STPs, industrial STPs, untreated sewage, hospital wastewater and direct dumping of unsegregated wastes. Effluent from a common effluent treatment plant (CETP) (Patancheru, Hyderabad, South India) receiving pharmaceutical wastewater from 90 bulk drug manufacturing companies was reported to carry very high concentrations (all following concentrations in µg L^-1^) of ciprofloxacin (14,000), enrofloxacin (210), lomefloxacin (8.8), norfloxacin (25), ofloxacin (55) and trimethoprim (4.4) (Fick et al., 2009). The effluent is discharged into Isakuvagu and Nakkavagu rivers (**Figure 6**). Analysing the water samples confirmed the presence of the same antibiotics as in the effluents, namely ciprofloxacin (10–2,500), enrofloxacin (BDL–30), lomefloxacin (BDL–1.1), norfloxacin (BDL–4.7), ofloxacin (BDL–4), and trimethoprim (BDL–4). Interestingly, enoxacin (BDL–66) was also detected despite its absence in the effluent of the CETP, which could be attributed to collecting samples on different days. Nageswara Rao et al. (2008) also found norfloxacin (0 0.024 ± 9.7), sulfamethoxazole (0.076 ± 6.8) and trimethoprim (0.087 ± 5.6) in the Nakkavagu river. Moreover, water samples from the lakes, tanks and ground water from the region were also reported to have various concentrations of the antibiotics. The antibiotics enoxacin (14–84), enrofloxacin (BDL–5), norfloxacin (60–520), ofloxacin (0.18–10), and trimethoprim (BDL–4) were detected in lake samples. Tanks contained ciprofloxacin (3–7), enoxacin (96–160), enrofloxacin (10–25), norfloxacin (91–200), and ofloxacin (5–7) and the ground water samples contained ciprofloxacin (0.18–14), enoxacin (0.08–2), enrofloxacin (BDL–0.067), norfloxacin (BDL–0.031), ofloxacin (BDL–0.48), and trimethoprim (BDL–0.055). Nageswara Rao et al. (2008) reported norfloxacin (0.048 ± 4.8) and sulfamethoxazole (0.047 ± 9.2) in Hussain Sagar Lake and ciprofloxacin (0.072 ± 8.4) and sulfamethoxazole (0.096 ± 4.3) in Kazipalli tank. However, antibiotic concentrations in the tap water and drinking water from this region were below detection limits (Fick et al., 2009). Nevertheless, antibiotics such as sulfamethoxazole (0.0007–0.034) and sulfanilamides (0.0068–0.12) have been reported in 26 ground water samples from urban regions (North India) (Lapworth et al., 2018). Velpandian et al. (2018) also reported several antibiotics of classes penicillins, fluoroquinolones, macrolides, and aminoglycosides in ground water across Delhi National Capital Region (North India) (**Table 5**). Glorian et al. (2018) studied antibiotic concentrations in the surface waters and riverbank filtration wells across selected sites in Uttarakhand, New Delhi and Uttar Pradesh (North India) along the Rivers Ganga and Yamuna. Five sampling campaigns conducted in September 2015, May and September 2016, September 2017, and June 2018 reported azithromycin, clarithromycin, erythromycin, roxithromycin and sulfamethoxazole at varying concentrations. The concentration of these antibiotics was relatively higher in samples collected from sites in New Delhi and Uttar Pradesh when compared to Uttarakhand. This difference could be attributed to regional variation in antibiotic consumption, ratio of population density to river water volume (could be lower in Uttarakhand) or dilution of sewage in receiving water bodies.

**Table 5:** Concentration of antibiotics observed in 35 samples of ground water collected at depths 12–46 m across Delhi National Capital Region (NCR) (North India) (Velpandian et al., 2018).

Antibiotic	Class of Antibiotic	Concentration (µg L^-1^)
**Amoxicillin**	Penicillin	0.18 ± 0.16
**Ciprofloxacin**	Fluoroquinolone	5.90 ± 4.04
**Ofloxacin**	Fluoroquinolone	4.34 ± 6.25
**Azithromycin**	Macrolide	0.17 ± 0.17
**Erythromycin**	Macrolide	0.11 ± 0.03
**Moxifloxacin**	Fluoroquinolone	0.21 ± 0.28
**Norfloxacin**	Fluoroquinolone	0.05 ± 0.04
**Sparfloxacin**	Fluoroquinolone	0.00 ± 0.00
**Kanamycin**	Aminoglycosides	0.06 ± 0.05
**Tobramycin**	Aminoglycosides	0.00 ± 0.00
**Streptomycin**	Aminoglycosides	0.24 ± 0.06
**Amikacin**	Aminoglycosides	0.00 ± 0.00
**Netlimycin**	Aminoglycosides	0.21 ± 0.07
**Neomycin**	Aminoglycosides	1.00 ± 0.29
**Gentamicin**	Aminoglycosides	0.06 ± 0.04

### 4.3 Antibiotics in soils

The use of antibiotics in agriculture and aquaculture is prevalent in India. Several studies suggest increasing AMR prevalence in bacteria isolated from poultry, other livestock and aquaculture sources as a result of excessive use. Antibiotics such as oxytetracycline, streptomycin, penicillin, oxolinic acid, and gentamycin are used in crop protection. Oxytetracycline, ampicillin, enrofloxacin, ciprofloxacin and sparfloxacin are widely used to promote growth and as prophylaxis in poultry and other livestock, and aquaculture. In aquaculture, these antibiotics are used as a means to increase survival of larvae. Last resort human antibiotics such as cephalosporins (3^rd^ generation) and colistin are used in swine farms, poultry, and cattle farms (Lekagul et al., 2018; Walia et al., 2019; Mann et al., 2021). The occurrence of veterinary antibiotics in soils and aquatic systems is predominantly due to their usage in agriculture, aquaculture and/or livestock settings. Antibiotics administered to animals are mostly excreted, and the application of this animal waste for soil fertilization often results in unintentional dispersion of antibiotics in soil and eventual dispersion to other environmental matrices. However, factors such as degradation rate, adsorption and solubility of antibiotics combined with soil properties and environmental conditions such as rainfall determine the extent of dispersion. In addition, untreated and treated wastewater, and sludge from STPs is widely used to fertilize soil (Minhas et al., 2022). During rain, the run-off water carries topsoil along with the antibiotics that ultimately reach surface water bodies (Manyi-Loh et al., 2018; Velpandian et al., 2018). In addition, unsegregated waste from households and hospitals containing antibiotics finds its way to landfills. Research suggests that unsegregated waste is one of the important sources of antibiotics in surface waters and aquifers. A study by Velpandian et al. (2018) reported 14 antibiotics of different classes in surface waters (Yamuna River, Delhi) and in 48 aquifers across Delhi, with high concentrations of antibiotics observed in aquifers close to a landfill site. Antibiotics such as ofloxacin (0.05–190) and trimethoprim (0.168) have been reported in leachate collected from landfills of Ghazipur (North India) and Coimbatore (South India), respectively (Subedi et al., 2015; Velpandian et al., 2018). Arun et al. (2022) also reported high levels of ciprofloxacin (up to 0.05 µg L^-1^), azithromycin (0.018 µg L^-1^) and sulfamethazine (0.052 µg L^-1^) in the ground water samples of Chennai, a metropolitan city in South India thought to be influenced by Kodungayur solid waste dumpsite (Arun et al., 2022) These studies emphasize the importance of appropriate disposal of unused and expired antibiotics from households and hospitals because dissolution of tablets could lead to locally higher concentrations of antibiotics, which may select for resistance as discussed in the introduction or may be toxic to more sensitive groups of organisms such as cyanobacteria (Tell et al., 2019). Continuous discharge of antibiotics could also result in accumulation leading to concentrations high enough to select.

## 5 Potential implications of antibiotic contamination

The pervasive contamination of water bodies with antibiotics could pose environmental and health challenges. Depending on concentrations, the presence of antibiotics in aquatic ecosystems could disrupt microbial communities and harm other aquatic organisms. For instance, studies have shown that exposure to certain antibiotics can alter fish physiology, which could ultimately lead to human health risks (Limbu et al., 2021). The risk to human health from consuming fish exposed to antibiotics primarily concerns the ingestion of antibiotic residues, which can exceed safe levels. Long-term exposure to low levels of antibiotics through diet could contribute to the development of antibiotic resistance and other adverse health effects. Moreover, antibiotic residues in water sources can enter the human food chain, potentially risking human health (Polianciuc et al., 2020), depending on the antibiotic and its concentration, which is typically much lower than those used in medical treatments. As such, the risk to human health from consumption of such residues is likely lower compared to the health risks associated with direct antibiotic treatment. In India, the issue is particularly pressing due to the extensive use of antibiotics and the presence of numerous pharmaceutical industries. Case studies from various regions in India that we have summarized here have demonstrated the presence of antibiotic residues in rivers and lakes, highlighting the urgent need for action (Taneja and Sharma, 2019).

Antibiotic resistance (AMR) in polluted water bodies is a growing concern, especially in densely populated countries like India. Numerous studies have documented the emergence of antibiotic-resistant bacteria in Indian water bodies, often correlating with high levels of antibiotic contamination (Global Antibiotic Resistance Partnership, 2011). However, such correlations do not prove causation as it is likely that the co-occurrence of antibiotics and antibiotic resistant bacteria in water bodies is due to a common cause, such as contamination by sewage or manure as these are sources for both. Most studies are not designed to be able to distinguish antibiotic selection for resistance in the sampled water body from co-occurrence due to pollution from the same source. Indeed, the relationship between antibiotic concentrations and the development of resistance is complex and influenced by various factors, including the concentrations and types of antibiotics and the characteristics of the microbial community and the environmental conditions present, as briefly explained in the introduction.

While the One Health approach is gaining acceptance in addressing antibiotic resistance, it is essential to apply quantitative reasoning to understand the strength and relevance of the interconnectedness among human, animal, and environmental health. Not all connections are equally important, and discerning the more impactful ones is crucial for effective strategies (One Health Initiative, 2020). To combat antibiotic resistance, the One Health approach involves several key strategies. These include expanding and enhancing wastewater treatment processes to reduce resistant microorganisms and antibiotic residues in water bodies, implementing stricter regulations on the use of antibiotics in healthcare and agriculture, and improving surveillance systems for monitoring antibiotic resistance patterns, but also improving WASH and access to antibiotics when needed to treat infections and preventing further spread (Aslam et al., 2021; OECD, 2023). In many cases, antibiotics are not available when needed, which can exacerbate the spread of infections. This lack of access necessitates a balanced approach in the One Health strategy, which not only focuses on reducing resistance through wastewater treatment but also on ensuring fair access to antibiotics for treating infections. India’s National Action Plan (NAP) for AMR has been launched to address antibiotic resistance through a One Health framework. These efforts are aligned with global strategies, like the World Health Organization’s Global Action Plan on Antimicrobial Resistance, which emphasizes the need for coordinated action across sectors and countries (Ranjalkar and Chandy, 2019). It’s crucial to recognize that the presence of antibiotics in the environment does not automatically lead to the selection for resistance. This process is dependent on various factors, including the concentration of antibiotics and the Minimum Selective Concentration (MSC), which is still a subject of ongoing research. Throughout this manuscript, care has been taken not to assert a direct causal relationship where the evidence is still emerging or uncertain.

## 6 Standard discharge limits and their economic impact

A zero liquid discharge (ZLD) policy is recommended for bulk drug and formulation industries in India (Environment (Protection) Amendment Rules, 2020). ZLD replaces discharge of effluent with wastewater treatment for reuse while reducing contaminants to solid waste. Reuse of treated effluents for gardening or horticulture is not considered as ZLD. Any solid wastes arising from ZLD are hazardous and should be transported under strict vigilance to an engineered land fill site. In January 2020, the Ministry of Environment, Forest and Climate Change, Government of India (GOI) amended the standard discharge limits for the effluents from bulk drug and formulation industries by including limits for 121 antibiotics in draft regulations published in the Gazette (Environment (Protection) Amendment Rules, 2020), see **Supplementary File S1** for a list of thresholds. Importantly, the concentration limits were to apply to the effluents before discharge into receiving rivers, which would have been substantially more stringent. However, concentration limits could be met by dilution of the effluent, and it is the mass loading to the receiving river that determines concentrations in the river. In addition, sludges containing antibiotics were required to be incinerated in a hazardous waste incinerator. The concentration thresholds for the antibiotics were ~2.5-fold lower than the predicted no-effect concentrations (PNECs) recommended by the AMR industry alliance (**Supplementary File S1**). The latter PNEC values were the lower value of MIC-based PNECs (Bengtsson-Palme and Larsson, 2016) and toxicological PNECs (Tell et al., 2019). India was thus the first country to propose concentration limits for antibiotics in 2020. However, in 2021, the Ministry of Environment, Forest and Climate Change dropped all limits for antibiotic concentrations in the effluent of bulk drug and formulation manufacturers from the final rules published in the Gazette (Environmental (Protection) Second Amendment Rules, 2021) see also **Supplementary File S1**. Removing these limits may have been due to fears that several Indian bulk drug manufacturers could be wiped out of the supply chain due to the stringent limits and that the cost of antibiotics would rise, although the expected additional cost arising from expensive LC-MS/MS analysis and additional wastewater treatment or ZLD process implementation to meet the limits has been estimated to be small (Welcome Trust and Boston Consulting Group, 2022). Financial compensation for companies adopting ZLD should be considered. To monitor compliance economically and rapidly, portable antibiotic sensors have recently been developed (Mathai et al., 2023).

## 7 An attempt to quantify the scarcity of the data

India, with its vast expanse and geological, biological, and climatic diversity, is naturally endowed with numerous water bodies that sustain all forms of life and a population of 1.4 billion people. It houses a staggering 2,424,540 water bodies with 97.1% in rural areas. Of these, 59.5% are ponds, followed by tanks (15.7%), reservoirs (12.1%), water conservation schemes/percolation tanks/check dams (9.3%), lakes *sensu stricto* (0.9%), and others (2.5%), as per the water bodies census by the Government of India Ministry of Jal Shakti Department of water resources river development (2023) which combines the Department of Water Resources, River Development and Ganga Rejuvenation & the Department of Drinking Water and Sanitation. Out of this huge number of water bodies, very few have been studied. Among the studies analysed, 20 focus on rivers, 12 on STPs, 6 on lakes and ponds, and 5 on groundwater. By adding up the studies conducted on rivers (20), lakes and ponds (6), and groundwater (5), we get a total of 31 studies. When compared to the staggering total of 2,424,540 water bodies in India, these studies represent approximately 0.0013% of all water bodies, indicating a significant gap in research coverage (**Figure 6**).

India has eight major river systems and over 400 rivers in total, of which 13 distinct rivers have been studied, a proportion of 13/400×100≈3.25%. Given the diverse environmental conditions that characterize different parts of India, the few rivers studied are unlikely to be representative. The Yamuna and Ganga, both iconic and of paramount significance to India’s socio-cultural and environmental fabric, have been amongst the most studied rivers. Nevertheless, even they have been poorly covered. For the Yamuna, four separate studies have been conducted with a total of 31 sampling points. If these were spread evenly across its length of 1376 km, each sampling point would represent an approximate stretch of 1376/31≈44.4 km. For the Ganga, three studies have sampled 27 distinct points. Distributing these points evenly across its length of 2525 km, each sampling point would represent an approximate stretch of 2525/27≈93.5 km. Considering the dynamic nature of river ecosystems and the large number of point sources of pollution, this is hardly sufficient.

## 8 Conclusions and future perspectives

This review has highlighted the significant challenges that antibiotic pollution and the risk of subsequent development of AMR presents to India, and consequently to the world. While some headway has been made in identifying the sources, pathways and sinks of antibiotics and influences on their distribution in India’s water environments, huge gaps remain. These include very few study locations as well as very infrequent sampling and a lack of coordination and standardization that resulted in different studies measuring different sets of antibiotics at different times in different ways. Scarce and hard to compare data is a problem that is of course not unique to India, but the situation in India can serve to illustrate the problem.

Clearly, it is impossible to monitor all rivers, let alone all water bodies, sufficiently frequently and with appropriate spatial resolution. We would like to propose a solution to this problem. The research community, possibly supported by citizen scientists, should identify two representative study sites for every combination of key factors influencing antibiotic concentrations (and likewise AMR): (1) population density (low, medium, high) and (2) size of water bodies (small, medium, large; for lakes in terms of liquid retention times, for rivers in terms of discharge) for (3) each geographic/climatic region of India. Additionally, it is crucial to incorporate the state of waste treatment facilities into this framework since their efficiency and coverage impacts the levels of contaminants in water bodies, including antibiotics. This will provide a more comprehensive understanding of the sources and dissemination pathways of antibiotics and AMR, thereby strengthening the proposed solution and enhancing our ability to effectively monitor and mitigate these environmental issues. Population density can be assumed to correlate with frequency of antibiotic consumption and thus antibiotic loading of point sources. Size of water bodies will affect how much any input from point sources will be diluted. As an illustration, a large city polluting a small river is expected to lead to high concentrations of antibiotics in rivers while a small city discharging pollution into a large river will have little effect – these would be the extremes. Geography and climate will affect the retention times and discharges. Hydrological modelling based on topography, land use and climate data could be used to provide reasonable estimates for these figures. The chosen study sites should then be sampled at high spatial and temporal resolution to estimate spatial and temporal variability and to determine whether the resolution can be reduced or should be increased in the future. Results from these study sites are meant to be representative – this must of course be tested on select new study sites and if found wanting, more refined combinations of factors or additional factors will have to be identified. Once the search for representative sites has been successful, monitoring of antibiotic pollution could be focused on these representative sites. This would enable coverage of the whole country with limited resources.

Another – complementary – way to make monitoring more feasible is the further development and use of handheld sensors to measure antibiotic concentrations in the field. With limited training, citizen scientists could use these to monitor sites with suspected high pollution.

While antibiotic pollution – and more broadly AMR – is a global issue, it is particularly acute in countries like India, where antibiotic use is widespread, and environmental monitoring and regulation can be challenging. Continued research and concerted efforts from government, industry, and civil society are crucial in addressing these challenges. More research is needed to understand the ecological risk and the risk of selection for resistance by mixtures of antibiotics and co-selective chemicals at low concentrations in complex microbial communities. This should lead to evidence-based environmental standards, but standards alone are not effective if they are not enforced. Raising awareness of the problem will be an important way to push for standards and their enforcement.

To counter antibiotic pollution, the three key areas for intervention are improved treatment of community waste and wastewaters, roll out of zero liquid discharge for antibiotic producers and reducing antibiotic consumption by humans and animals. Only about 1/5 of wastewater is currently treated in STPs that meet discharge regulations. Increasing STP capacity in urban areas and decentralized wastewater management in rural areas is clearly needed and would remove not only antibiotics but also resistant bacteria and pathogens. In addition, improving the effectiveness of STPs in removing antibiotics, other pharmaceuticals and bacteria from the effluents would make an important contribution as currently, STPs only remove a fraction of these. New technologies are in development and should be optimized further to reduce energy consumption and cost. Antibiotic production has led to very high concentrations of antibiotics in the environment surrounding production facilities. The risk of selection for more effective resistance and new combinations of resistance at these high concentrations is clearly substantial, therefore, zero liquid discharge should be applied in all production facilities. Last but not least, reducing antibiotic consumption by humans and animals is crucial yet complex and challenging. Use of antibiotics is appropriate to treat infections and thus prevent further spread of the infections that could ultimately require the use of more antibiotics. Use of antibiotics to treat viral infections should be avoided. Moreover, prevention of infections is better than treatment. This should include improved waste management, supply of clean water, nutrition, hygiene, and vaccinations. Increased public awareness will again be important to drive change.

The issue of antibiotic pollution is a serious one, but it is one that is not insurmountable. By taking steps to better understand the risks and to develop effective solutions, we can help to protect the environment, animals, and public health.

## Author contributions

SR: Conceptualization, Formal analysis, Methodology, Writing – original draft, Writing – review & editing. CU: Conceptualization, Formal Analysis, Methodology, Writing – original draft, Writing – review & editing. VS: Conceptualization, Formal analysis, Methodology, Writing – review & editing. ST: Formal analysis, Funding acquisition, Supervision, Writing – review & editing. IN: Formal analysis, Funding acquisition, Supervision, Writing – review & editing. JK: Conceptualization, Formal analysis, Funding acquisition, Supervision, Writing – original draft, Writing – review & editing.

## References

[B1] Andersson D. I. Levin B. R. (1999). The biological cost of antibiotic resistance. Curr. Opin. Microbiol. 2, 489–493. doi: 10.1016/S1369-5274(99)00005-3 10508723

[B2] Andersson D. I. Hughes D. (2011). Persistence of antibiotic resistance in bacterial populations. FEMS Microbiol Rev. 35, 901–911. doi: 10.1111/j.1574-6976.2011.00289.x 21707669

[B3] Antimicrobial Resistance Collaborators Murray C. J. Ikuta K. S. Sharara F. Swetschinski L. Robles Aguilar G.. (2022). Global burden of bacterial antimicrobial resistance in 2019: a systematic analysis. Lancet 399, 629–655. doi: 10.1016/S0140-6736(21)02724-0 35065702 PMC8841637

[B4] Archana G. Dhodapkar R. Kumar A. (2016). Offline solid-phase extraction for preconcentration of pharmaceuticals and personal care products in environmental water and their simultaneous determination using the reversed phase high-performance liquid chromatography method. Environ. Monit Assess. 188, 512. doi: 10.1007/s10661-016-5510-1 27502523

[B5] Arun S. Xin L. Gaonkar O. Neppolian B. Zhang G. Chakraborty P. (2022). Antibiotics in sewage treatment plants, receiving water bodies and groundwater of Chennai city and the suburb, South India: Occurrence, removal efficiencies, and risk assessment. Sci. Total Environ. 851, 158195. doi: 10.1016/j.scitotenv.2022.158195 35995170

[B6] Aslam B. Khurshid M. Arshad M. I. Muzammil S. Rasool M. Yasmeen N.. (2021). Antibiotic resistance: one health one world outlook. Front. Cell Infect. Microbiol. 11. doi: 10.3389/fcimb.2021.771510 PMC8656695 34900756

[B7] Baquero F. Martínez J. L. Cantón R. (2008). Antibiotics and antibiotic resistance in water environments. Curr. Opin. Biotechnol. 19, 260–265. doi: 10.1016/j.copbio.2008.05.006 18534838

[B8] Bengtsson-Palme J. Larsson D. G. J. (2016). Concentrations of antibiotics predicted to select for resistant bacteria: Proposed limits for environmental regulation. Environ. Int. 86, 140–149. doi: 10.1016/j.envint.2015.10.015 26590482

[B9] Berendonk T. U. Manaia C. M. Merlin C. Fatta-Kassinos D. Cytryn E. Walsh F.. (2015). Tackling antibiotic resistance: The environmental framework. Nat. Rev. Microbiol. 13, 310–317. doi: 10.1038/nrmicro3439 25817583

[B10] Biswas P. Vellanki B. P. (2021). Occurrence of emerging contaminants in highly anthropogenically influenced river Yamuna in India. Sci. Total Environ. 782, 146741. doi: 10.1016/j.scitotenv.2021.146741 33839659

[B11] Blanchard J. Solaipandian M. John E. B. Pandith M. Jeo B. Saji S.. (2021). Self-prescribing of antibiotics by patients seeking care in Indian emergency departments. JACEP Open 2, e12432. doi: 10.1002/emp2.12432 33969344 PMC8082699

[B12] Calderón C. Sabundayo B. P. (2007). “Antimicrobial classifications: drugs for bugs,” in Antimicrobial Susceptibility Testing Protocols. Eds. Schwalbe R. Steele-Moore L. Goodwin A. C. (CRC Press), 7–53.

[B13] Carvalho I. T. Santos L. (2016). Antibiotics in the aquatic environments: A review of the European scenario. Environ. Int. 94, 736–757. doi: 10.1016/j.envint.2016.06.025 27425630

[B14] Central Pollution Control Board (2017). Consolidated annual review report on implementation of solid wastes management rules.

[B15] Central Pollution Control Board (2021) National Inventory of Sewage Treatment Plants (Parivesh Bhawan East Arjun Nagar). Available at: https://cpcb.nic.in/openpdffile.php?id=UmVwb3J0RmlsZXMvMTIyOF8xNjE1MTk2MzIyX21lZGlhcGhvdG85NTY0LnBkZg (Accessed December 28, 2023).

[B16] Chandy S. J. Thomas K. Mathai E. Antonisamy B. Holloway K. A. Stalsby Lundborg C. (2013). Patterns of antibiotic use in the community and challenges of antibiotic surveillance in a lower-middle-income country setting: A repeated cross-sectional study in Vellore, South India. J. Antimicrobial Chemotherapy 68, 229–236. doi: 10.1093/jac/dks355 22945913

[B17] Cisneros-Mayoral S. Graña-Miraglia L. Perez-Morales D. Peña-Miller R. Fuentes-Hernández A. (2022). Evolutionary history and strength of selection determine the rate of antibiotic resistance adaptation. Mol. Biol. Evol. 39, msac185. doi: 10.1093/molbev/msac185 36062982 PMC9512152

[B18] Delafuente J. Toribio-Celestino L. Santos-Lopez A. León-Sampedro R. Alonso-Del Valle A. Costas C.. (2022) Within-patient evolution of plasmid-mediated antimicrobial resistance Europe PMC Funders Group. Available at: https://www.springernature.com/gp/open-research/policies/accepted-manuscript-terms. 10.1038/s41559-022-01908-7 PMC7613874 36303001

[B19] Diwan V. Hanna N. Purohit M. Chandran S. Riggi E. Parashar V.. (2018). Seasonal variations in water-quality, antibiotic residues, resistant bacteria and antibiotic resistance genes of escherichia coli isolates from water and sediments of the kshipra river in central India. Int. J. Environ. Res. Public Health 15, 1281. doi: 10.3390/ijerph15061281 29914198 PMC6024939

[B20] Diwan V. Stålsby Lundborg C. Tamhankar A. J. (2013). Seasonal and temporal variation in release of antibiotics in hospital wastewater: estimation using continuous and grab sampling. PloS One 8, e68715. doi: 10.1371/journal.pone.0068715 23861936 PMC3704537

[B21] Diwan V. Tamhankar A. J. Khandal R. K. Sen S. Aggarwal M. Marothi Y.. (2010). Antibiotics and antibiotic-resistant bacteria in waters associated with a hospital in Ujjain, India. BMC Public Health (2010) 10, 414. doi: 10.1186/1471-2458-10-414 PMC2912816 20626873

[B22] Environment (Protection) Amendment Rules (2020). The Gazette of India-Ministry of Environment, Forest and Climate Change Notification.

[B23] Environmental (Protection) Second Amendment Rules (2021). The Gazette of India-Ministry of Environment, Forest and Climate Change Notification.

[B24] Fang P. Elena A. X. Kunath M. A. Berendonk T. U. Klümper U. (2023). Reduced selection for antibiotic resistance in community context is maintained despite pressure by additional antibiotics. ISME Commun. 3, 1–9. doi: 10.1038/s43705-023-00262-4 PMC10232432 37258727

[B25] Fick J. Söderström H. Lindberg R. H. Phan C. Tysklind M. Larsson D. G. J. (2009). Pharmaceuticals and personal care products in the environment contamination of surface, ground, and drinking water from pharmaceutical production. Environ. Toxicol. Chem. 28, 2522–2527. doi: 10.1897/09-073.S1 19449981

[B26] Finley R. L. Collignon P. Larsson D. G. J. Mcewen S. A. Li X. Z. Gaze W. H.. (2013). The scourge of antibiotic resistance: The important role of the environment. Clin. Infect. Dis. 57, 704–710. doi: 10.1093/cid/cit355 23723195

[B27] Gandra S. Joshi J. Trett A. Sankhil Lamkang A. (2017). Scoping Report on antimicrobial resistance in India.

[B28] Global Antibiotic Resistance Partnership (2011) Rationalizing antibiotic use to limit antibiotic resistance in India + Global Antibiotic Resistance Partnership (GARP)-India Working Group* Status Report. Available at: www.cddep.org/publications.

[B29] Glorian H. Börnick H. Sandhu C. Grischek T. (2018). Water quality monitoring in northern India for an evaluation of the efficiency of bank filtration sites. Water (Basel) 10, 1804. doi: 10.3390/w10121804

[B30] Gopal C. M. Bhat K. Praveenkumarreddy Y. Shailesh Kumar V. Basu H.. (2020). Evaluation of selected pharmaceuticals and personal care products in water matrix using ion trap mass spectrometry: A simple weighted calibration curve approach. J. Pharm. BioMed. Anal. 185, 113214. doi: 10.1016/j.jpba.2020.113214 32126444

[B31] Gopal C. M. Bhat K. Ramaswamy B. R. Kumar V. Singhal R. K. Basu H.. (2021). Seasonal occurrence and risk assessment of pharmaceutical and personal care products in Bengaluru rivers and lakes, India. J. Environ. Chem. Eng. 9, 105610. doi: 10.1016/j.jece.2021.105610

[B32] Gothwal R. Shashidhar (2017a). Occurrence of high levels of fluoroquinolones in aquatic environment due to effluent discharges from bulk drug manufacturers. J. Hazard Toxic Radioact Waste 21, 05016003. doi: 10.1061/(asce)hz.2153-5515.0000346

[B33] Gothwal R. Shashidhar T. (2017b). Proliferation of ciprofloxacin resistant bacteria in polluted sediments of musi river, India. Soil Sediment Contam 26, 501–509. doi: 10.1080/15320383.2017.1355352

[B34] Government of India Ministry of Jal Shakti Department of water resources river development (2023). “Ganga rejuvenation minor irrigation (statistics) wing,” in Water Bodies First Census Report, vol. 1.

[B35] Greulich P. Scott M. Evans M. R. Allen R. J. (2015). Growth-dependent bacterial susceptibility to ribosome-targeting antibiotics. Mol. Syst. Biol. 11, 796. doi: 10.15252/msb.20145949 26146675 PMC4380930

[B36] Gullberg E. Cao S. Berg O. G. Ilbäck C. Sandegren L. Hughes D.. (2011). Selection of resistant bacteria at very low antibiotic concentrations. PloS Pathog. 7, 3313. doi: 10.1371/journal.ppat.1002158 PMC3141051 21811410

[B37] Hanna N. Purohit M. Diwan V. Chandran S. P. Riggi E. Parashar V.. (2020). Monitoring of Water Quality, Antibiotic Residues, and Antibiotic-Resistant Escherichia coli in the Kshipra River in India over a 3-Year Period. Int. J. Environ. Res. Public Health 17, 7706. doi: 10.3390/ijerph17217706 33105585 PMC7659961

[B38] Harrison E. Guymer D. Spiers A. J. Paterson S. Brockhurst M. A. (2015). Parallel compensatory evolution stabilizes plasmids across the parasitism-mutualism continuum. Curr. Biol. 25, 2034–2039. doi: 10.1016/j.cub.2015.06.024 26190075

[B39] Harrower J. McNaughtan M. Hunter C. Hough R. Zhang Z. Helwig K. (2021). Chemical fate and partitioning behavior of antibiotics in the aquatic environment—A review. Environ. Toxicol. Chem. 40, 3275–3298. doi: 10.1002/etc.5191 34379810

[B40] Hassoun-Kheir N. Stabholz Y. Kreft J. U. de la Cruz R. Dechesne A. Smets B. F.. (2021). EMBRACE-WATERS statement: Recommendations for reporting of studies on antimicrobial resistance in wastewater and related aquatic environments. One Health 13, 100339. doi: 10.1016/j.onehlt.2021.100339 34746357 PMC8554267

[B41] Hassoun-Kheir N. Stabholz Y. Kreft J. U. de la Cruz R. Romalde J. L. Nesme J.. (2020). Comparison of antibiotic-resistant bacteria and antibiotic resistance genes abundance in hospital and community wastewater: A systematic review. Sci. Total Environ. 743, 140804. doi: 10.1016/j.scitotenv.2020.140804 32758846

[B42] Jackson A. R. Eismont A. Yu L. Li N. Gu W. Eismont F.. (2018). Diffusion of antibiotics in intervertebral disc. J. Biomech 76, 259–262. doi: 10.1016/j.jbiomech.2018.06.008 29941209 PMC6082158

[B43] Jha R. R. Singh N. Kumari R. Patel D. K. (2017). Ultrasound-assisted emulsification microextraction based on a solidified floating organic droplet for the rapid determination of 19 antibiotics as environmental pollutants in hospital drainage and Gomti river water. J. Sep Sci. 40, 2694–2702. doi: 10.1002/jssc.201700170 28474761

[B44] Joshua D. I. Praveenkumarreddy Y. Prabhasankar V. P. D’Souza A. P. Yamashita N. Balakrishna K. (2020). First report of pharmaceuticals and personal care products in two tropical rivers of southwestern India. Environ. Monit Assess. 192, 529. doi: 10.1007/s10661-020-08480-2 32681316 PMC7367900

[B45] Kapoor G. Saigal S. Elongavan A. (2017). Action and resistance mechanisms of antibiotics: A guide for clinicians. J. Anaesthesiol Clin. Pharmacol. 33, 300–305. doi: 10.4103/joacp.JOACP_349_15 29109626 PMC5672523

[B46] Kotwani A. Joshi J. Lamkang A. S. (2021). Over-the-counter sale of antibiotics in India: A qualitative study of providers’ perspectives across two states. Antibiotics 10, 1123. doi: 10.3390/antibiotics10091123 34572705 PMC8472180

[B47] Koya S. F. Ganesh S. Selvaraj S. Wirtz V. J. Galea S. Rockers P. C. (2022). Consumption of systemic antibiotics in India in 2019. Lancet Reg. Health Southeast Asia (2022) 4, 100025. doi: 10.1016/j PMC10305917 37383993

[B48] Kraupner N. Hutinel M. Schumacher K. Gray D. A. Genheden M. Fick J.. (2021). Evidence for selection of multi-resistant E. coli by hospital effluent. Environ. Int. 150, 106436. doi: 10.1016/j.envint.2021.106436 33592450

[B49] Kümmerer K. (2009). Antibiotics in the aquatic environment - A review - Part I. Chemosphere 75, 417–434. doi: 10.1016/j.chemosphere.2008.11.086 19185900

[B50] Kurasam J. Mandal P. K. Sarkar S. (2022). Selective proliferation of antibiotic-resistant bacteria in the biological treatment process at a municipal wastewater treatment plant in India. J. Environ. Eng. 148, 04022007. doi: 10.1061/(asce)ee.1943-7870.0001980

[B51] Lapworth D. J. Das P. Shaw A. Mukherjee A. Civil W. Petersen J. O.. (2018). Deep urban groundwater vulnerability in India revealed through the use of emerging organic contaminants and residence time tracers. Environ. pollut. 240, 938–949. doi: 10.1016/j.envpol.2018.04.053 29949845

[B52] Larsson D. G. J. de Pedro C. Paxeus N. (2007). Effluent from drug manufactures contains extremely high levels of pharmaceuticals. J. Hazard Mater 148, 751–755. doi: 10.1016/j.jhazmat.2007.07.008 17706342

[B53] Laxminarayan R. Chaudhury R. R. (2016). Antibiotic resistance in India: drivers and opportunities for action. PloS Med. 13, e1001974. doi: 10.1371/journal.pmed.1001974 26934098 PMC4775002

[B54] Lekagul A. Tangcharoensathien V. Yeung S. (2018). The use of antimicrobials in global pig production: A systematic review of methods for quantification. Prev. Vet. Med. 160, 85–98. doi: 10.1016/j.prevetmed.2018.09.016 30389002

[B55] Limbu S. M. Chen L. Q. Zhang M. L. Du Z. Y. (2021). A global analysis on the systemic effects of antibiotics in cultured fish and their potential human health risk: a review. Rev. Aquac 13, 1015–1059. doi: 10.1111/raq.12511

[B56] Loftie-Eaton W. Bashford K. Quinn H. Dong K. Millstein J. Hunter S.. (2017). Compensatory mutations improve general permissiveness to antibiotic resistance plasmids. Nat. Ecol. Evol. 1, 1354–1363. doi: 10.1038/s41559-017-0243-2 29046540 PMC5649373

[B57] Lübbert C. Baars C. Dayakar A. Lippmann N. Rodloff A. C. Kinzig M.. (2017). Environmental pollution with antimicrobial agents from bulk drug manufacturing industries in Hyderabad, South India, is associated with dissemination of extended-spectrum beta-lactamase and carbapenemase-producing pathogens. Infection 45, 479–491. doi: 10.1007/s15010-017-1007-2 28444620

[B58] Manaia C. M. Rocha J. Scaccia N. Marano R. Radu E. Biancullo F.. (2018). Antibiotic resistance in wastewater treatment plants: Tackling the black box. Environ. Int. 115, 312–324. doi: 10.1016/j.envint.2018.03.044 29626693

[B59] Mann A. Nehra K. Rana J. S. Dahiya T. (2021). Antibiotic resistance in agriculture: Perspectives on upcoming strategies to overcome upsurge in resistance. Curr. Res. Microb. Sci. 2, 100030. doi: 10.1016/j.crmicr.2021.100030 34841321 PMC8610298

[B60] Manyi-Loh C. Mamphweli S. Meyer E. Okoh A. (2018). Antibiotic use in agriculture and its consequential resistance in environmental sources: potential public health implications. Molecules 23, 795. doi: 10.3390/molecules23040795 29601469 PMC6017557

[B61] Mathai T. Pal T. Prakash N. Mukherji S. (2023). Portable biosensor for the detection of Enrofloxacin and Ciprofloxacin antibiotic residues in food, body fluids, environmental and wastewater samples. Biosens Bioelectron 237, 115478. doi: 10.1016/j.bios.2023.115478 37356410

[B62] Minhas P. S. Saha J. K. Dotaniya M. L. Sarkar A. Saha M. (2022). Wastewater irrigation in India: Current status, impacts and response options. Sci. Total Environ. 808, 152001. doi: 10.1016/j.scitotenv.2021.152001 34856275

[B63] Mohapatra S. Huang C.-H. Mukherji S. Padhye L. P. (2016). Occurrence and fate of pharmaceuticals in WWTPs in India and comparison with a similar study in the United States. Chemosphere 159, 526–535. doi: 10.1016/j.chemosphere.2016.06.047 27341156

[B64] Mutiyar P. K. Mittal A. K. (2013). Occurrences and fate of an antibiotic amoxicillin in extended aeration-based sewage treatment plant in Delhi, India: a case study of emerging pollutant. Desalination Water Treat 51, 6158–6164. doi: 10.1080/19443994.2013.770199

[B65] Mutiyar P. K. Mittal A. K. (2014). Occurrences and fate of selected human antibiotics in influents and effluents of sewage treatment plant and effluent-receiving river Yamuna in Delhi (India). Environ. Monit Assess. 186, 541–557. doi: 10.1007/s10661-013-3398-6 24085621

[B66] Nageswara Rao R. Venkateswarlu N. Narsimha R. (2008). Determination of antibiotics in aquatic environment by solid-phase extraction followed by liquid chromatography–electrospray ionization mass spectrometry. J. Chromatogr A 1187, 151–164. doi: 10.1016/j.chroma.2008.02.021 18295772

[B67] OECD (2023). Embracing a One Health Framework to Fight Antimicrobial Resistance.

[B68] One Health Initiative (2020) One Health (One Health Initiative). Available at: http://www.onehealthinitiative.com (Accessed December 28, 2023).

[B69] Page M. J. McKenzie J. E. Bossuyt P. M. Boutron I. Hoffmann T. C. Mulrow C. D.. (2021). The PRISMA 2020 statement: an updated guideline for reporting systematic reviews. BMJ, n71. doi: 10.1136/bmj.n71 33782057 PMC8005924

[B70] Pérez-Rodríguez F. Mercanoglu Taban B. (2019). A state-of-art review on multi-drug resistant pathogens in foods of animal origin: risk factors and mitigation strategies. Front. Microbiol. 10. doi: 10.3389/fmicb.2019.02091 PMC6742700 31555256

[B71] Polianciuc S. I. Gurzău A. E. Kiss B. Ștefan M. G. Loghin F. (2020). Antibiotics in the environment: causes and consequences. Med. Pharm. Rep. doi: 10.15386/mpr-1742 PMC7418837 32832887

[B72] Prabhasankar V. P. Joshua D. I. Balakrishna K. Siddiqui I. F. Taniyasu S. Yamashita N.. (2016). Removal rates of antibiotics in four sewage treatment plants in South India. Environ. Sci. pollut. Res. 23, 8679–8685. doi: 10.1007/s11356-015-5968-3 26797959

[B73] PubChem Amoxicillin (2023) PubChem Amoxicillin Summary (National Center for Biotechnology). Available at: https://pubchem.ncbi.nlm.nih.gov/compound/33613 (Accessed December 28, 2023).

[B74] PubChem Azithromycin (2023) PubChem Azithromycin Summary (National Center for Biotechnology). Available at: https://pubchem.ncbi.nlm.nih.gov/compound/447043 (Accessed December 28, 2023).

[B75] PubChem Ciprofloxacin (2023) PubChem Ciprofloxacin Summary (National Center for Biotechnology). Available at: https://pubchem.ncbi.nlm.nih.gov/compound/2764 (Accessed December 28, 2023).

[B76] PubChem Compound Summary (2023) PubChem Compound Summary (National Center for Biotechnology). Available at: https://pubchem.ncbi.nlm.nih.gov/ (Accessed December 28, 2023).

[B77] PubChem Doxycycline (2023) PubChem Doxycycline Summary (National Center for Biotechnology). Available at: https://pubchem.ncbi.nlm.nih.gov/compound/54671203 (Accessed December 28, 2023).

[B78] PubChem Erythromycin (2023) PubChem Erythromycin Summary (National Center for Biotechnology). Available at: https://pubchem.ncbi.nlm.nih.gov/compound/12560 (Accessed December 28, 2023).

[B79] PubChem Gentamicin (2023) PubChem Gentamicin Summary (National Center for Biotechnology). Available at: https://pubchem.ncbi.nlm.nih.gov/compound/3467 (Accessed December 28, 2023).

[B80] PubChem Metronidazole (2023) PubChem Metronidazole Summary (National Center for Biotechnology). Available at: https://pubchem.ncbi.nlm.nih.gov/compound/4173 (Accessed December 28, 2023).

[B81] PubChem Sulfamethoxazole (2023) PubChem Sulfamethoxazole Summary (National Center for Biotechnology). Available at: https://pubchem.ncbi.nlm.nih.gov/compound/5329 (Accessed December 28, 2023).

[B82] PubChem Tetracycline (2023) PubChem Tetracycline Summary (National Center for Biotechnology). Available at: https://pubchem.ncbi.nlm.nih.gov/compound/54675776 (Accessed December 28, 2023).

[B83] PubChem Trimethoprim (2023) PubChem Trimethoprim Summary (National Center for Biotechnology). Available at: https://pubchem.ncbi.nlm.nih.gov/compound/5578 (Accessed December 28, 2023).

[B84] PubChem Vancomycin (2023). PubChem Vancomycin Summary (National Center for Biotechnology. Available at: https://pubchem.ncbi.nlm.nih.gov/compound/14969. (Accessed December 28, 2023).

[B85] Quintela-Baluja M. Frigon D. Abouelnaga M. Jobling K. Romalde J. L. Gomez Lopez M.. (2021). Dynamics of integron structures across a wastewater network – Implications to resistance gene transfer. Water Res. 206, 117720. doi: 10.1016/j.watres.2021.117720 34673462 PMC8626773

[B86] Ranjalkar J. Chandy S. (2019). India’s National Action Plan for antimicrobial resistance – An overview of the context, status, and way ahead. J. Family Med. Prim Care 8, 1828. doi: 10.4103/jfmpc.jfmpc_275_19 31334140 PMC6618210

[B87] Renganathan J. S I. U. H. Ramakrishnan K. Ravichandran M. K. Philip L. (2021). Spatio-temporal distribution of pharmaceutically active compounds in the River Cauvery and its tributaries, South India. Sci. Total Environ. 800, 149340. doi: 10.1016/j.scitotenv.2021.149340 34399341

[B88] Rodriguez-Mozaz S. Chamorro S. Marti E. Huerta B. Gros M. Sànchez-Melsió A.. (2015). Occurrence of antibiotics and antibiotic resistance genes in hospital and urban wastewaters and their impact on the receiving river. Water Res. 69, 234–242. doi: 10.1016/j.watres.2014.11.021 25482914

[B89] Saxena P. Hiwrale I. Das S. Shukla V. Tyagi L. Pal S.. (2021). Profiling of emerging contaminants and antibiotic resistance in sewage treatment plants: An Indian perspective. J. Hazard Mater 408, 124877. doi: 10.1016/j.jhazmat.2020.124877 33383454

[B90] Serwecińska L. (2020). Antimicrobials and antibiotic-resistant bacteria: A risk to the environment and to public health. Water (Switzerland) 12. doi: 10.3390/w12123313

[B91] Sharma B. M. Bečanová J. Scheringer M. Sharma A. Bharat G. K. Whitehead P. G.. (2019). Health and ecological risk assessment of emerging contaminants (pharmaceuticals, personal care products, and artificial sweeteners) in surface and groundwater (drinking water) in the Ganges River Basin, India. Sci. Total Environ. 646, 1459–1467. doi: 10.1016/j.scitotenv.2018.07.235 30235631

[B92] Shimizu A. Takada H. Koike T. Takeshita A. Saha M. Rinawati. (2013). Ubiquitous occurrence of sulfonamides in tropical Asian waters. Sci. Total Environ. 452–453, 108–115. doi: 10.1016/j.scitotenv.2013.02.027 23500404

[B93] Singh K. P. Rai P. Singh A. K. Verma P. Gupta S. (2014). Occurrence of pharmaceuticals in urban wastewater of north Indian cities and risk assessment. Environ. Monit Assess. 186, 6663–6682. doi: 10.1007/s10661-014-3881-8 25004851

[B94] Singh V. Suthar S. (2021). Occurrence, seasonal variations, and ecological risk of pharmaceuticals and personal care products in River Ganges at two holy cities of India. Chemosphere 268, 129331. doi: 10.1016/j.chemosphere.2020.129331 33359991

[B95] Stanton I. C. Murray A. K. Zhang L. Snape J. Gaze W. H. (2020). Evolution of antibiotic resistance at low antibiotic concentrations including selection below the minimal selective concentration. Commun. Biol. 3, 1–11. doi: 10.1038/s42003-020-01176-w PMC7471295 32884065

[B96] Subedi B. Balakrishna K. Joshua D. I. Kannan K. (2017). Mass loading and removal of pharmaceuticals and personal care products including psychoactives, antihypertensives, and antibiotics in two sewage treatment plants in southern India. Chemosphere 167, 429–437. doi: 10.1016/j.chemosphere.2016.10.026 27750166

[B97] Subedi B. Balakrishna K. Sinha R. K. Yamashita N. Balasubramanian V. G. Kannan K. (2015). Mass loading and removal of pharmaceuticals and personal care products, including psychoactive and illicit drugs and artificial sweeteners, in five sewage treatment plants in India. J. Environ. Chem. Eng. 3, 2882–2891. doi: 10.1016/j.jece.2015.09.031

[B98] Taneja N. Sharma M. (2019). Antimicrobial resistance in the environment: The Indian scenario. Indian J. Med. Res. 149, 119–128. doi: 10.4103/ijmr.IJMR_331_18 31219076 PMC6563737

[B99] Tell J. Caldwell D. J. Häner A. Hellstern J. Hoeger B. Journel R.. (2019). Science-based targets for antibiotics in receiving waters from pharmaceutical manufacturing operations. Integr. Environ. Assess. Manag 15, 312–319. doi: 10.1002/ieam.4141 30884149 PMC6849714

[B100] Van Boeckel T. P. Gandra S. Ashok A. Caudron Q. Grenfell B. T. Levin S. A.. (2014). Global antibiotic consumption 2000 to 2010: An analysis of national pharmaceutical sales data. Lancet Infect. Dis. 14, 742–750. doi: 10.1016/S1473-3099(14)70780-7 25022435

[B101] Velpandian T. Halder N. Nath M. Das U. Moksha L. Gowtham L.. (2018). Un-segregated waste disposal: an alarming threat of antimicrobials in surface and ground water sources in Delhi. Environ. Sci. pollut. Res. 25, 29518–29528. doi: 10.1007/s11356-018-2927-9 30136185

[B102] Vogwill T. Maclean R. C. (2015). The genetic basis of the fitness costs of antimicrobial resistance: A meta-analysis approach. Evol. Appl. 8, 284–295. doi: 10.1111/eva.12202 25861386 PMC4380922

[B103] Walia K. Sharma M. Vijay S. Shome B. (2019). Understanding policy dilemmas around antibiotic use in food animals & offering potential solutions. Indian J. Med. Res. 149, 107. doi: 10.4103/ijmr.IJMR_2_18 31219075 PMC6563746

[B104] Welcome Trust and Boston Consulting Group (2022). Understanding the antibiotic manufacturing ecosystem A view of global supply chains, pressure points, and implications for antimicrobial resistance response.

[B105] Wilkinson J. L. Boxall A. B. A. Kolpin D. W. Leung K. M. Y. Lai R. W. S. Galbán-Malagón C.. (2022). Pharmaceutical pollution of the world’s rivers. Proc. Natl. Acad. Sci. 119, e2113947119. doi: 10.1073/pnas.2113947119 35165193 PMC8872717

[B106] Williams M. Kookana R. S. Mehta A. Yadav S. K. Tailor B. L. Maheshwari B. (2019). Emerging contaminants in a river receiving untreated wastewater from an Indian urban centre. Sci. Total Environ. 647, 1256–1265. doi: 10.1016/j.scitotenv.2018.08.084 30180334

[B107] World Health Organization (2014). Antimicrobial resistance global report on surveillance.

