# Supplementary material for: Mapping the scarcity of data on antibiotics in natural and engineered water environments across India
Source: Front Antibiot. 2024 Feb 12;3:1337261. doi: 10.3389/frabi.2024.1337261 (PMC11732091; doi:10.3389/frabi.2024.1337261)
Supplement: Supplementary file 1 [file DataSheet_1.zip › Supplementary Tables 1 and 2.docx]

Supplementary Material

**Mapping the Scarcity of Data on Antibiotics in Natural and Engineered Water Environments Across India**

**Sasikaladevi Rathinavelu^1,2^** ^†^ ***, Cansu Uluseker^2^** ^†^**^*^, Vikas Sonkar^3^, Shashidhar Thatikonda^3^, Indumathi M Nambi^1^, Jan Ulrich-Kreft^2^ ***

^1^Environmental Engineering Division, Department of Civil Engineering, Indian Institute of Technology Madras, Chennai, India

^2^School of Biosciences & Institute of Microbiology and Infection, University of Birmingham, Birmingham, United Kingdom

^3^IIT Hyderabad, Kandi, Sangareddy, Medak District, Telangana, India

*** Correspondence:** j.kreft@bham.ac.uk, c.uluseker@bham.ac.uk, sasikaladevi.rathinavelu@eawag.ch

**Includes:**

**Supplementary Table 1**. Concentrations of antibiotics observed during winter, rainy, and summer seasons in two hospital effluents.

**Supplementary Table 2**. Concentrations of antibiotics reported in influent, effluent, and sludge of STPs in Southern and Northern India employing an Activated Sludge Process (ASP).

**Data S1** (Excel format). Comparison of Indian Environment Protection Rules with the recommendations of the AMR Industry Alliance antibiotic discharge targets “List of predicted no-effect concentrations (PNECs)” 2018

**Supplementary Table 1.** Average concentration of antibiotics (µg L^-1^) reported during in two hospital effluents collected during winter, rainy, and summer seasons (Diwan et al., 2013). These data are displayed in Figure 3 in the main text. Note composite and grab samples can be quite different.

Samples: Composite sample (CS), Grab sample (GS)

Antibiotics: Ciprofloxacin (CIP), Levofloxacin (LVX), Ofloxacin (OFX), Norfloxacin (NOR), Fluoroquinolones (FQs), Metronidazole (MTZ), Sulfamethoxazole (SMX)

| **Antibiotics →** | **CIP** | | **LVX** | | **OFX** | | **NOR** | | **FQs** | | **MTZ** | | **SMX** | |
| --- | --- | --- | --- | --- | --- | --- | --- | --- | --- | --- | --- | --- | --- | --- |
| **Season↓** | **CS** | **GS** | **CS** | **GS** | **CS** | **GS** | **CS** | **GS** | **CS** | **GS** | **CS** | **GS** | **CS** | **GS** |
| Summer | 0.1555 | – | – | 0.472 | 0.035 | 0.096 | – | – | 0.191 | 0.568 | 0.131 | 0.018 | 0.034 | 1.174 |
| Rainy | 0.694 | 1.239 | 0.066 | 0.088 | 0.09 | 0.085 | 0.04 | 0.225 | 0.891 | 1.638 | 0.145 | 0.143 | 0.56 | 0.076 |
| Winter | 0.245 | 1.836 | 0.578 | 1.078 | 0.495 | 0.475 | – | – | 1.318 | 3.389 | 0.036 | 0.018 | 0.106 | 0.355 |

**Supplementary Table 2**. Concentrations of antibiotics reported in influent, effluent, and sludge of STPs in Southern and Northern India employing an Activated Sludge Process (ASP)

References: (^1^Arun et al., 2022; ^2^Kurasam et al., 2022; ^3,4^Subedi et al., 2015, 2017)

N.D. -Not Detected

Antibiotics: Azithromycin (AZM), Ciprofloxacin (CIP), Clindamycin (CLI), Enrofloxacin (ENO), Erythromycin (ERY), Lincomycin (LIN), Norfloxacin (NOR), Ofloxacin (OFX), Roxithromycin (RXT), Sulfamethazine (SMT), Sulfamethoxazole (SMX) and Trimethoprim (TMP)

| Region | STP Location | Sample | Concentration Water (µg L^-1^), Sludge (µg g^-1^) | | | | | | | | | | | |
| --- | --- | --- | --- | --- | --- | --- | --- | --- | --- | --- | --- | --- | --- | --- |
|  |  |  | AZM | CIP | CLI | ENO | ERY | LIN | NOR | OFX | RXT | SMT | SMX | TMP |
| Northern | **Saidpur, Bihar ^3,4^** | Influent |  |  | 0.00516 |  |  | 0.0152 |  |  |  |  | 0.195 | 0.033 |
|  |  | Effluent |  |  | 0.048 |  |  | 0.053 |  |  |  |  | N.D. | 0.0348 |
|  |  | Sludge |  |  | 0.00203 |  |  | 0.00085 |  |  |  |  | N.D. | N.D. |
|  | **Beur, Bihar ^3,4^** | Influent |  |  | 0.0183 |  |  | 0.0208 |  |  |  |  | 0.288 | 0.0908 |
|  |  | Effluent |  |  | 0.00696 |  |  | 0.0175 |  |  |  |  | 0.0702 | 0.038 |
|  |  | Sludge |  |  | 0.00672 |  |  | 0.00177 |  |  |  |  | 0.031 | N.D. |
|  | **Haridwar, Uttarakhand^2^** | Influent |  | 13.6 |  |  |  |  | 11.1 | 7.7 |  |  |  |  |
|  |  | Effluent |  | 5 |  |  |  |  | 1 | 2.5 |  |  |  |  |
| Southern | **Udupi, Karnataka ^3,4^** | Influent |  |  | 0.0496 |  |  | 0.226 |  |  |  |  | 0.414 | 0.16 |
|  |  | Effluent |  |  | 0.0638 |  |  | 0.187 |  |  |  |  | 0.228 | N.D. |
|  |  | Sludge |  |  | 0.0806 |  |  | 0.0473 |  |  |  |  | N.D. | N.D. |
|  | **Coimbatore, Tamil Nadu ^3,4^** | Influent |  |  | 0.0272 |  |  | N.D. |  |  |  |  | 0.552 | 0.156 |
|  |  | Effluent |  |  | 0.0175 |  |  | 0.00392 |  |  |  |  | 0.318 | 0.103 |
|  |  | Sludge |  |  | 0.0485 |  |  | 0.0015 |  |  |  |  | N.D. | N.D. |
|  | **Manipal, Karnataka ^3,4^** | Influent |  |  | 1.87 |  |  | 0.148 |  |  |  |  | 2.26 | 0.0356 |
|  |  | Effluent |  |  | 0.952 |  |  | 0.043 |  |  |  |  | 0.296 | 2.08 |
|  | **Chennai, Tamil Nadu ^1^** | Influent | 0.00117–0.01087 | 0.00685–0.1453 |  | 0.00021–0.0008 | N.D.– 0.06217 |  | N.D.– 0.0573 | N.D.– 0.1626 | N.D.– 0.00051 | N.D.– 0.0026 | N.D.– 0.0492 | N.D.– 0.0038 |
|  |  | Effluent | N.D.–0.0026 | 0.0073–0.09 |  | 0.0002–0.0009 | N.D.– 0.0033 |  | N.D.– 0.0368 | N.D.– 0.1329 | N.D. | N.D.– 0.0004 | N.D.– 0.0122 | N.D.– 0.0028 |

**References**

Arun, S., Xin, L., Gaonkar, O., Neppolian, B., Zhang, G., & Chakraborty, P. (2022). Antibiotics in sewage treatment plants, receiving water bodies and groundwater of Chennai city and the suburb, South India: Occurrence, removal efficiencies, and risk assessment. *Science of the Total Environment*, *851*(158195 Contents). https://doi.org/10.1016/j.scitotenv.2022.158195

Diwan, V., Lundborg, S. S., & Tamhankar, C. J. (2013). Seasonal and Temporal Variation in Release of Antibiotics in Hospital Wastewater: Estimation Using Continuous and Grab Sampling. *PLoS ONE*, *8*(7), 68715. https://doi.org/10.1371/journal.pone.0068715

Kurasam, J., Mandal, P. K., & Sarkar, S. (2022). Selective Proliferation of Antibiotic-Resistant Bacteria in the Biological Treatment Process at a Municipal Wastewater Treatment Plant in India. *Journal of Environmental Engineering*, *148*(4), 1–10. https://doi.org/10.1061/(asce)ee.1943-7870.0001980

Subedi, B., Balakrishna, K., Joshua, D. I., & Kannan, K. (2017). Mass loading and removal of pharmaceuticals and personal care products including psychoactives, antihypertensives, and antibiotics in two sewage treatment plants in southern India. *Chemosphere*, *167*, 429–437. https://doi.org/10.1016/j.chemosphere.2016.10.026

Subedi, B., Balakrishna, K., Sinha, R. K., Yamashita, N., Balasubramanian, V. G., & Kannan, K. (2015). Mass loading and removal of pharmaceuticals and personal care products, including psychoactive and illicit drugs and artificial sweeteners, in five sewage treatment plants in India. *Journal of Environmental Chemical Engineering*, *3*(Part 4A), 2882–2891. https://doi.org/10.1016/j.jece.2015.09.031
